# Deep Eutectic Solvents as Convenient Media for Synthesis of Novel Coumarinyl Schiff Bases and Their QSAR Studies

**DOI:** 10.3390/molecules22091482

**Published:** 2017-09-05

**Authors:** Maja Molnar, Mario Komar, Harshad Brahmbhatt, Jurislav Babić, Stela Jokić, Vesna Rastija

**Affiliations:** 1 Faculty of Food Technology Osijek, Josip Juraj Strossmayer University of Osijek, Franje Kuhaca 20, Osijek 31000, Croatia; mmolnar@ptfos.hr (M.M.); mario.komar@ptfos.hr (M.K.); brahmbhattharshad@hotmail.com (H.B.); jurislav.babic@ptfos.hr (J.B.); stela.jokic@ptfos.hr (S.J.); 2 Faculty of Agriculture in Osijek, Josip Juraj Strossmayer University of Osijek, Vladimira Preloga 1, Osijek 31000, Croatia

**Keywords:** coumarin, schiff base, deep eutectic solvents, antioxidant activity, QSAR

## Abstract

Deep eutectic solvents, as green and environmentally friendly media, were utilized in the synthesis of novel coumarinyl Schiff bases. Novel derivatives were synthesized from 2-((4-methyl-2-oxo-2*H*-chromen-7-yl)oxy)acetohydrazide and corresponding aldehyde in choline chloride:malonic acid (1:1) based deep eutectic solvent. In these reactions, deep eutectic solvent acted as a solvent and catalyst as well. Novel Schiff bases were synthesized in high yields (65–75%) with no need for further purification, and their structures were confirmed by mass spectra, ^1^H and ^13^C NMR. Furthermore, their antioxidant activity was determined and compared to antioxidant activity of previously synthesized derivatives, thus investigating their structure–activity relationship utilizing quantitative structure-activity relationship QSAR studies. Calculation of molecular descriptors has been performed by DRAGON software. The best QSAR model (*R*_tr_ = 0.636; *R*_ext_ = 0.709) obtained with three descriptors (*MATS3m*, *Mor22u*, *Hy*) implies that the pairs of atoms higher mass at the path length 3, three-dimensional arrangement of atoms at scattering parameter *s* = 21 Å^−1^, and higher number of hydrophilic groups (-OH, -NH) enhanced antioxidant activity. Electrostatic potential surface of the most active compounds showed possible regions for donation of electrons to 1,1-diphenyl-2-picryhydrazyl (DPPH) radicals.

## 1. Introduction

Coumarins are a class of compounds widely distributed in the plant kingdom [1], but lots of synthetic studies have been done on them in the last few decades. Most of the synthetic modifications aim at the synthesis of biologically active derivatives with more potent specific biological activity and their potential application in pharmacy, cosmetic industry or medicine. Different antioxidants have been synthesized and investigated by many researchers in this regard. We have been investigating the antioxidant activity of different synthetic coumarin derivatives for years and showed that different synthetic modifications on the basic coumarin core can increase its antioxidant activity [2,3,4,5]. For this purpose, a series of Schiff bases were synthesized [2] conventionally; however, we have noticed their formation when deep eutectic solvents (DESs) are applied as well. DESs have proven to be a convenient media for many synthetic routes and are often characterized as environmentally friendly [6,7,8]. Their application in organic synthesis and extraction, as well as an extensive analysis of their properties, were described in some good reviews published in the last few years [6,7,8,9,10,11].

As the emphasis is put on green chemistry approaches on a daily basis, “green solvents” are the focus of many researchers, since the most waste that is produced in the synthesis of different compounds are actually waste solvents [12]. DESs are an emerging class of new, non-toxic, biodegradable solvents, which have already found their application in organic synthesis, and they are becoming more prominent every day, since they have many advantages compared to conventional solvents. Capua et al. [12] synthesized 2-aminoimidazoles in one-pot protocol in high yields and with very simple post synthetic workup, in shorter times than with conventional organic solvents like tetrahydrofuran (THF), and this procedure resulted in no by-products, while DES was recovered and recycled. Massolo et al. [13] employed DESs in enantioselective transformations, also obtaining products at high yields with the possibility of DESs being recycled and reused, and the same advantages of DESs were noticed in the synthesis of imines and hydrobenzamides [14] and enantioselective aldol reaction [15]. DESs have proven to be effective in umpolung sulfenylation reactions [16], applicable in material science in epoxy resin polymerization [17], and as catalysts in CO_2_ fixation epichlorohydrin [18].

Due to DESs’ numerous advantages compared to organic solvents, the aim of this study was to synthesize novel coumarinyl Schiff bases utilizing choline chloride based DES as reaction media and catalyst as well. The fact that there was no need for a specific catalyst, like acetic acid, which is often used in these kinds of reactions, was noticed. Not only did we get a novel compounds, but we also applied an environmentally friendly approach, utilizing a solvent made of biodegradable components, with low toxicity and vapor pressure, which can be easily recycled and reused [19]. The purpose of the study was also to determine their antioxidant activity and compare it to antioxidant activities of previously synthesized similar Schiff bases [2,5].

Many theoretical studies have showed that biological activity of natural and synthetic antioxidants is related to their structure [20]. Different quantitative structure–activity relationship (QSAR) techniques have been employed to correlate the antioxidant capacity with various structural features of coumarin derivatives that affect the antioxidant activity and predict the same for the untested and future molecules, such as multiple linear regression [21,22], genetic function approximation genetic partial least squares [23,24], and artificial neural networks [25]. Wide ranges of molecular descriptors, mathematical representations of a molecule, have been used for QSAR modeling of antioxidant activities of a series of coumarin derivatives. Thus, physicochemical properties such as hydrophilic factor [24,25] and lipophilicity [21]; energy based descriptors, such as bond dissociation enthalpy [26]; two-dimensional (2D) descriptors Moran autocorrelation and BCUT (Burden - CAS - University of Texas eigen values) descriptors and three-dimensional (3D) descriptors GATEWAY descriptors [27] showed the most significant influences on the QSAR modeling of antioxidant activity of coumarines. Accurate QSAR models have been generated using quantum-chemistry descriptors, such as HOMO (highest occupied molecular orbital), LUMO (lowest unoccupied molecular orbital), the difference of the energies HOMO-LUMO, and hardness [24,25,28,29].

QSAR models obtained for series of coumarin derivatives that were modeled for their antioxidant activity based on their ability to inhibit DPPH free radical, revealed that compounds with a higher degree of branching, oxygen atom-bearing fragment and tertiary carbon as substituent lie in the higher activity range [23]. QSAR study of 3-carboxycoumarin derivatives has implied an importance of the presence of hydroxyl groups on the ring structure and a higher number of hydrophilic groups for enhanced DPPH radical scavenging activity [25]. In this work, QSAR study was performed on the set of synthesized coumarin derivatives in order to signify the importance of structural and chemical attributes for their antioxidant activity.

## 2. Results

### 2.1. Synthesis

**Scheme 1:**
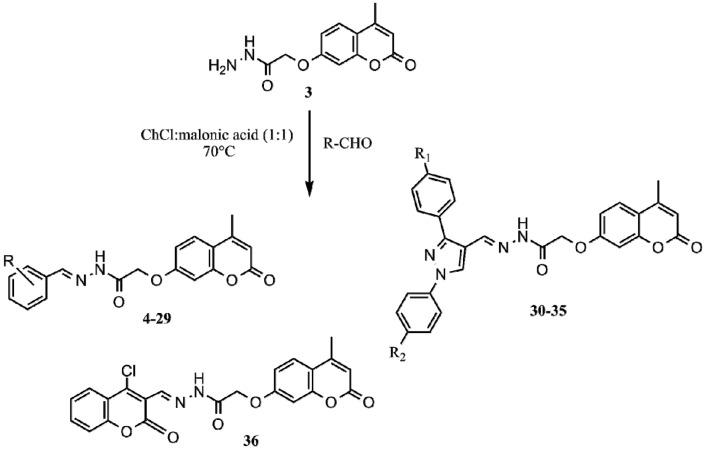
Synthesis of novel coumarinyl Schiff bases in deep eutectic solvent (DES).

**Scheme 2:**
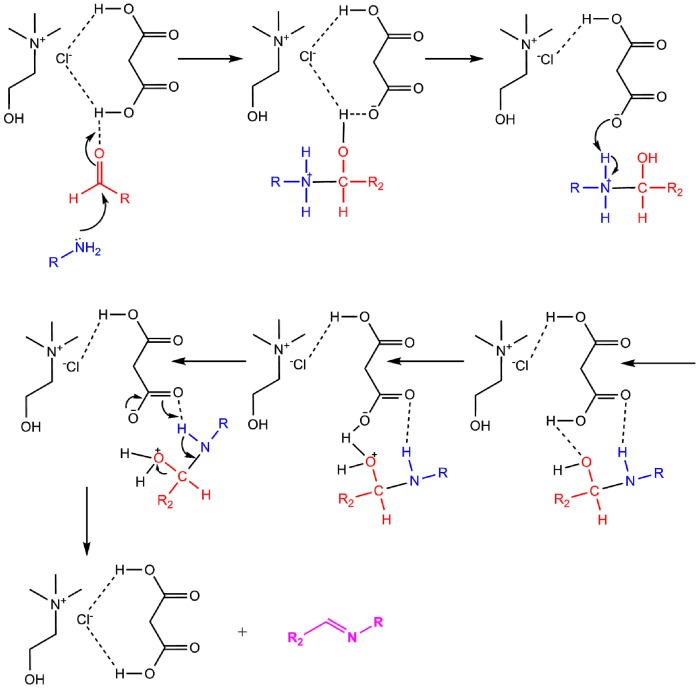
Proposed mechanism for synthesis of Schiff bases in choline chloride:malonic acid (1:1) DES.

Synthesis of Schiff bases is often performed by a reaction of amine with aldehyde, usually with the addition of catalysts such as acetic acid. Here, we describe a synthesis of Schiff bases from 2-((4-methyl-2-oxo-2*H*-chromen-7-yl)oxy)acetohydrazide and different aldehydes utilizing choline chloride:malonic acid DES. DES was easily prepared from choline chloride and malonic acid (1:1), simply by heating their mixture at 80 °C until clear liquid was formed and used, as such, without any further purification, in synthesis of desired Schiff bases. First, we performed reactions with some commercial aldehydes, 4-methoxybenzaldehyde and 4-dimethylaminobenzaldehyde, to obtain Schiff bases **10** and **29**, which have already been synthesized conventionally in our previous research [2,5]. The yields of compounds obtained conventionally (60% for compound **10** and 86% for compound **29** [5]) compared to those obtained in DES (44% for compound **10** and 70% for compound **29**) are not much higher, which justified the use of this environmentally acceptable approach in further synthesis of novel Schiff bases. Therefore, further synthesis was performed with some new aldehydes to obtain compounds **30**–**36** (Scheme 1). Pyrazole based aldehydes utilized for a synthesis of Schiff bases **30**–**35** were synthesized in a reaction of substituted acetophenones with different hydrazines and subsequent formylation to yield 1*H*-pyrazole-4-carbaldehyde. For synthesis of Schiff bases, choline chloride:malonic acid (1:1) DES was used as reaction media. The synthetic approach (Scheme 1) in the synthesis of mentioned Schiff bases in DES is rather simple. When DES is prepared, an equimolar ratio of 2-((4-methyl-2-oxo-2*H*-chromen-7-yl)oxy)acetohydrazide and aldehyde was added to the solvent, stirred at 70 °C and, upon completion of the reaction, water was added and a solid product separated. DESs were found to be very effective in this kind of synthesis, both, as solvents and catalysts, since reaction times were not longer than four hours, and purity of final compounds was satisfying as well as the yields. The mechanism of their action in accordance with Yadav et al. [30] is shown in Scheme 2.

Schiff bases were obtained in high yields (44–95%) and characterized by ^1^H- and ^13^C-NMR, as well as mass spectrometry. Typical shifts for coumarin C3 proton (6.20–6.22 ppm) and C4-methyl group (2.40 ppm) were noticed. The synthesized coumarin derivatives show characteristic peaks for aromatic protons, coumarin aromatic protons, protons of aromatic rings derived from corresponding aldehyde and pyrazole proton. All molecular masses were also in accordance with molecular ions obtained by mass spectrometry. The structures of synthesized compounds (**30**–**36**) are presented in Table 1. The ^1^H- and ^13^C-NMR spectra of synthesized compounds are presented in supplementary file 1 (File S1).

### 2.2. Antioxidant Activity

Novel compounds (**30**–**36**), together with Schiff bases synthesized in our previous work [2,5], were investigated for their antioxidant activity expressed as % DPPH scavenging activity.

Data presented in Table 1 show that substituents on the phenyl ring have a great influence on antioxidant activity. Compounds **1**–**3** were used as basic structures in antioxidant activity determination to demonstrate how modification of a starting compound can have a great influence on antioxidant activity. Compared to coumarin's basic structure (**1**), substitution in position 4 with methyl and in position 7 with hydroxy group enhances antioxidant activity (**2**). Substitution of 7-OH group with -CO-NH-NH_2_ group in 2-((4-methyl-2-oxo-2*H*-chromen-7-yl)oxy)acetohydrazide (**3**) additionally increased antioxidant activity.

Novel compounds, together with Schiff bases synthesized in our previous work [2,5] were investigated for their antioxidant activity expressed as % DPPH scavenging activity. Structural details of all studied molecules and % DPPH are shown in Table 1**.** Data presented in Table 1 show that substituents on the phenyl ring have a great influence on antioxidant activity. Compounds with two hydroxyl groups in *ortho* position (**11**,**14**) exhibited the best antioxidant activity, although hydroxyl groups at the 2,3-position (**11**) compared to the 3,4-position (**14**) showed enhanced antioxidant activity. Catehol structure or 3,4-diOH substitution of phenyl ring allows oxygen atoms to cause electron delocalization and stabilisation of phenoxyl radical [2,5,31]. Novel compounds (**30**–**35**) did not show significant antioxidant activity. Replacement of aromatic ring by pyrazole ring in these compounds reduced antioxidant power compared to compounds (**4**–**29**).

### 2.3. QSAR Models

The best QSAR model for antioxidant activity of 36 coumarinyl Schiff bases was obtained by DRAGON descriptors:

log % DPPH = 0.696 + 1.474 (0.488) *Mor22u* + 1.948 (0.398) *MATS3m* + 0.387 (0.398) *Hy* *N*(training) = 29; *N*(test) = 7 (**6**,**12**,**19**,**24,28**,**33**,**34**) (1)

**Figure 1:**
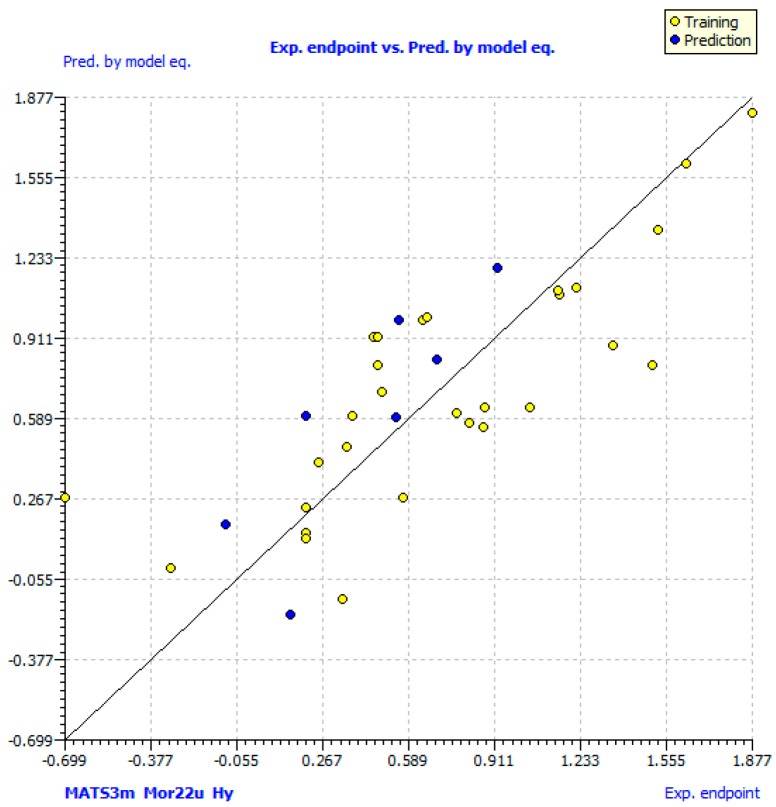
Observed versus predicted log % DPPH for the coumarinyl Schiff bases calculated by model (1).

Statistical parameters of obtained models are given in Table 2. The variables in Equation (1) are listed in order of relative importance by their standardized regression coefficients (β, in brackets). Table 3 presents a correlation matrix of descriptors included in model (1) and proves that descriptors are not mutually correlated (correlation coefficient, *R* ≤ 0.7). Low collinearity is also verified by the low values of *Kxx* and Δ*K* (≥0.05) [32] (Table 2). The molecular descriptor values; experimental and calculated log %DPPH by model (1) have been tabulated in supplementary file 2 (Table S2). A scatter plot of experimentally obtained antioxidant activity versus calculated by model (1) is shown in Figure 1.

**Figure 2:**
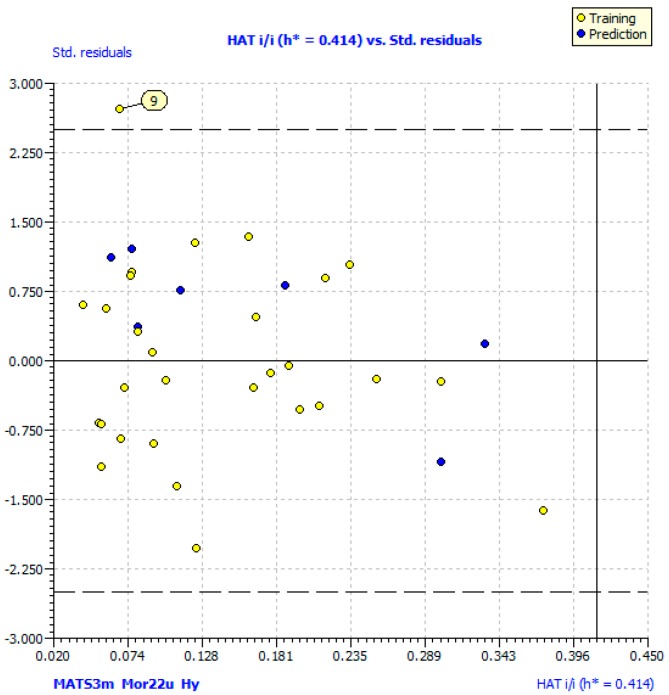
Williams plot of the applicability domain of the the QSAR model for antioxidant activity calculated by model (1).

According the statistical results presented in Table 2, model (1) satisfactoriness threshold for fitting validation parameters: *R*^2^ > 0.60 and value of *R*^2^_adj_ is close to the value of *R*^2^. The leave-one out (LOO) validation highlights that the model is stable, not obtained by chance, since *Q*^2^_LOO_ > 0.5; *R*^2^yscr, and *Q*^2^yscr < 0.2, as *R*^2^yscr > *Q*^2^yscr [33,34]. Moreover, the root-mean-square error (*RMSE)* values for the training and validation sets are similar. The chosen models demonstrate a satisfactory stability in external validation: *R*^2^_ext_ ≥ 0.60; small difference between *CCC*_tr_ and *CCC*_ext_; small difference between *RMSE*_tr_ and *RMSE*_ex_, and between mean absolute error of the training set (*MAE*_tr_) and mean absolute error of the external validation set *(MAE*_ex_) [35]. Parameter $\bar{r_{m}^{2}}$ considers the actual difference between the experimental and the predicted values, serving as more accurate measures for rating of model predictivity. Value of $\bar{r_{m}^{2}}>0.6$ confirms high prediction ability for both internal and external validation sets [36]. Williams plot (Figure 2) was used in order to define the chemical domain of applicability for which a given QSAR model makes reliable predictions [37]. Inspection of the Williams plot revealed no compounds out of hat value of leverage or warning leverage (HAT, h* = 0.414), which means that all predicted data for compounds belonging to the chemical domain and have reliable prediction. Williams plot identified one outlier (compound **9**), which has standardized residual predictions greater than 2.5 standard deviation units. Outlying behaviour of compound **9** was expected since it has demonstrated lowest antioxidant activity (Table 1). The methoxy group in phenyl ring, especially in position 3 (compound **9**), inactivates the ring, which negatively influences the ability of the compound to form a stable radical upon scavenging DPPH radicals [38].

After removal of the compound **9** from the training set, subsequent re-analysis produced a following improved QSAR model:

log % DPPH = 0.733 + 1.331 (0.495) *Mor22u* + 1.744 (0.401) *MATS3m* + 0.375 (0.375) *Hy* N(training) = 28; N(test) = 7 (**6,12,19,24,28,33,34**) (β in brackets) (2)

Calculated values of log %DPPH by model (2) have been tabulated in Table S2. Since compound **9** belonged to the training set, the obtained model gives the better statistics in terms of improvement in fitting and internal criteria. Exclusion of outliers generated the model with higher values of *R*^2^, *R*^2^_adj_, *F*, *CCC*_tr_, *CCC*_cv_, *Q*^2^_LOO_ and lower values of *s*, *RMSE*_tr_, *MAE*_tr_ RMSE_cv_, and MAE_cv_. The obtained model in external validation showed only improvement in *R*^2^_ext_, which demonstrated that the removal of outliers can improve the fitting, but not the predictivity of a model.

The largest values of standardized regression coefficients in Equations (1) and (2) has 3D-MoRSE (Molecular Representation of Structures based on Electronic diffraction) descriptor *Mor22u*. According the positive coefficients of *Mor22u* in Equations (1) and (2), compounds with significant antioxidant ability have enhanced positive values of this descriptor (Table S2). Descriptor *Mor22u* denotes unweighted descriptors with scattering parameter *s* = 21 Å^−1^. Since it is unweighted, the descriptor has no discriminative ability precisely and treats each atom equally. Though each 3D-MoRSE descriptor reflects the three-dimensional arrangement of the atoms in molecules, their final values are derived mostly from short distances [39]. Since mainly QSAR study covers structurally similar sets of compounds, 3D-MoRSE descriptors in a model can be interpreted using just several pairs of neighbor atoms. Specifically, descriptor *Mor22u* has the possibility to distinguish the difference between bond lengths of any kinds of atoms at least 0.03 Å [39]. The relative discriminative power of descriptor *Mor22u* can be observed at molecules **11**–**15**, which have two hydroxyl groups on phenyl rings (Table 1 and Table S2). This descriptor is extremely sensitive to the difference in di-OH substitution of the phenyl ring. The value of *Mor22u* for the most active compound **11** (log %DPPH = 1.817), which has two hydroxyl groups in position 2,3 is 0.3. Substitution of two OH groups in compound **12** at the position 2,4 decreased values of *Mor22u* (0.216) and antioxidant activity (log %DPPH = 0.924). Our recent study has also evinced sensitivity of the 3D-MoRSE descriptor *Mor19e* to the changes of substituents and their position on the 3,4-ethylenedioxythiophene central unit [40].

The 2D autocorrelation molecular descriptor, *MATS3m*, corresponds to the Moran autocorrelation descriptor –lag 3/weighted by atomic masses. The given descriptor describes how atomic mass is distributed along a topological molecular structure, precisely indicating dependence of one atom on value of mass through a topological structure of compounds [41]. Its positive regression coefficient in models (1) and (2) suggests that the increased number of pairs of atoms higher atomic mass at the path length 3 characterized compounds with enhanced antioxidant activity. The descriptor is sensitive to kind and position of substituents on the phenyl ring (Table 1 and Table S2). The best differences can be observed between compounds **11** and **14**, as well as **12** and **15**. Compounds bearing 2,3-dihydroxyphenyl (**11**) and 3,4-dihydroxyphenyl moieties (**14**) on benzene rings have two oxygen atoms at path length 3. Consequently, these compounds have higher values of *MATS3m* than compounds with 2,4-dihydroxyphenyl (**12**) and 3,5-dihydroxyphenyl (**15**) moieties, and exhibit higher antioxidant activity. Hence, descriptor *MATS3m* has ability to distinguish the structure with two hydroxyl groups in *ortho* position at the phenolic ring from structure with two hydroxyl groups in *meta* position, the structural requirements relevant for the effective radical scavenging of phenolic compounds.

The third variable in models (1) and (2) is hydrophilic index (*Hy*). Hydrophilic index takes into account a total number of hydrogen atoms attached to oxygen, sulfur and nitrogen atoms, and the number of carbon atoms in relation of number of non-hydrogen atoms [42]. Positive regression coefficients of *Hy* in both models indicate that higher number of -OH and -NH groups are favorable for antioxidant activity. This is expected since DPPH radicals become stable molecules by accepting hydrogen radicals from organic substances [43]. Since the hydrophilic factor can predict the ability of molecules to donate a hydrogen atom, it is an important descriptor in DPPH radical scavenging. Hydrophilic index counts only the number of hydrogen donating groups, and it is independent from their positions in molecules; therefore, its relative importance in models (1) and (2) is minimal. Hydrophilic index has been found in previous QSAR studies of the DPPH radical scavenging activity of coumarin derivatives. Results of those studies showed that incorporation of hydroxyl groups in coumarin molecules enhanced hydrophilic index, which increased DPPH radical scavenging effects [24,25].

### 2.4. Electrostatic Potential (ESP) Surface

**Figure 3:**
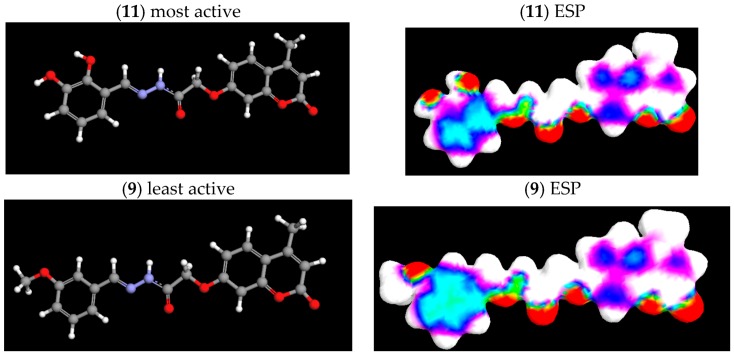

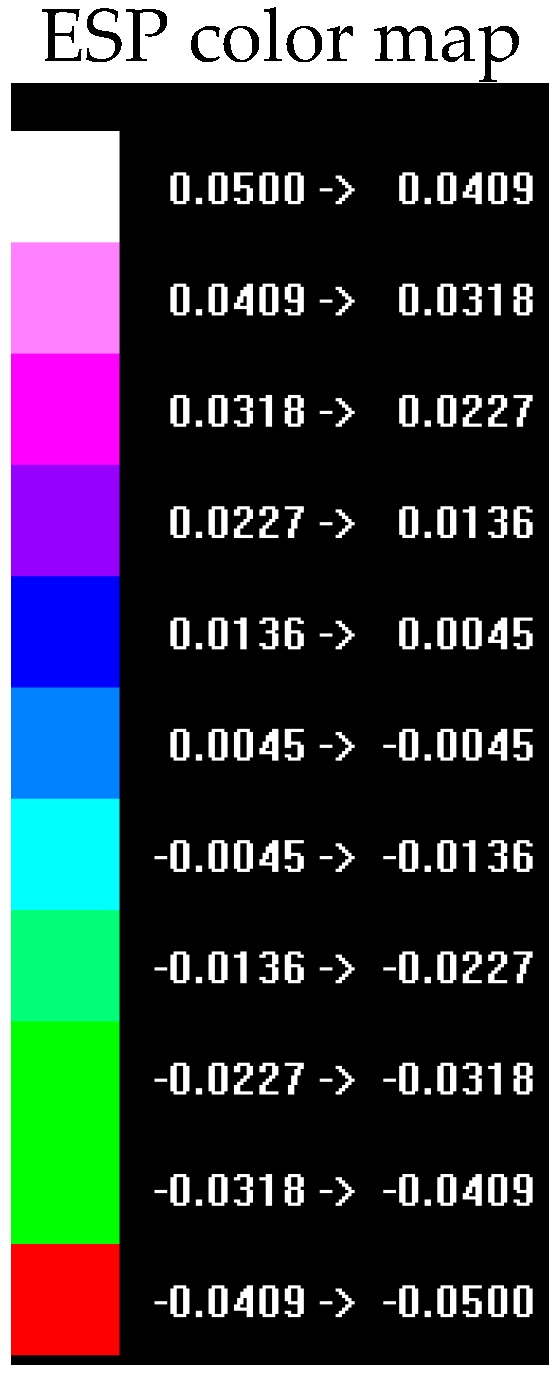
Three-dimensional optimized structure (MM+, AM1) and electrostatic potential (ESP) surface maps of the most active compound (**11**) and the least active compound (**9**).

Electrostatic potential (ESP) surface provides a visualization of total charge distribution of the molecule and relative polarity of the molecule [44]. Figure 3 presents an ESP mapped density surface of the most active molecule (**11**) and the least active molecule (**9**), for comparison. The red region shows the greatest increase in electron density or region of negative ESP. The light blue shows about zero, while the white color positive electron density. The negative regions (red color) correspond to the aggregation of electron density. The positive regions, which increase in order blue < violet < white color, indicate positive ESP that corresponds to the repulsion of the proton by the atomic nuclei or nucleophilic reactivity [45].

ESP maps of compounds (**9**) and (**11**) shows that the greatest negative electrostatic potential is located mainly over the oxygen and nitrogen from imino groups (>C=N-) with minimum value of −0.0409 au. A weakly positive region (from 0.0045 to 0.0318 au) is localized mostly on the carbon atoms of phenyl rings. Highly positive electron density is located on the hydrogen atoms. Observing ESP maps can help in the explanation of the role of substitution in antioxidant activity. Comparing the ESP maps of molecules, it could be observed that positive charge is more spread over the phenyl ring of inactive molecule (**9**). Negative charge of oxygen atoms from the 3-OCH_3_ group stabilizes positive charge, decreasing the ability for electron transfer to the DPPH radical. On the contrary, a smaller positive region in the phenyl ring of the most active molecule (**11**) allows easy electron transfer from negative charges located on two oxygen atoms from 2,3-OH groups.

## 3. Materials and Methods

### 3.1. Chemistry

All the chemicals were of p.a. purity and purchased from commercial suppliers. Melting points were determined on a capillary melting point apparatus (Electrothermal Engineering Ltd., Rochford, UK) and are uncorrected. Thin-layer chromatography was performed with fluorescent silica gel plates F254 (Merck, Darmstadt, Germany), under UV (254 and 365 nm) light, with benzene–acetone–acetic acid (8:1:1, *v*/*v*) as a solvent. The mass spectra were recorded on liquid chromatography tandem mass spectrometry (LC/MS/MS) API 2000 (Applied Biosystems/MDS SCIEX, Foster City, CA, USA). NMR spectra were recorded on a Bruker Avance 600 MHz NMR Spectrometer (Bruker Biospin GmbH, Rheinstetten, Germany) at 293 K in dimethylsulfoxide-*d*_6_ (DMSO-*d*_6_). The absorbance was measured on UV visible spectrophotometer Helios γ (ThermoSpectronic, Cambridge, UK).

### 3.2. Preparation of DES

DES was prepared as described previously [7]. Briefly, choline chloride and malonic acid were mixed together in molar ratio 1:1 and heated up to 80 °C until a clear liquid was obtained. This DES was used as such in synthesis of desired compounds as described in Section 3.3.

### 3.3. Synthesis of Schiff Bases in DES Choline Chloride:Malonic Acid (1:1)

An equimolar ratio of 2-((4-methyl-2-oxo-2*H*-chromen-7-yl)oxy)acetohydrazide and corresponding aldehyde was mixed in DES and stirred at 70 °C until the completion of reaction, which was monitored by thin-layer chromatography (TLC). Upon addition of water, a crude product was precipitated and washed with ethanol to obtain a pure Schiff base.

### 3.4. Characterization of Compounds

*(E)-N′-(4-Methoxybenzylidene)-2-((4-methyl-2-oxo-2H-chromen-7-yl)oxy)acetohydrazide* (**10**). Yield 44%; m.p. = 263 °C; ^1^H-NMR (ppm): 2.40 (s, 3H, CH_3_), 3.80 (s, 3H, OCH_3_), 4.79–5.27 (s, 2H, CH_2_), 6.22 (s, H, CH), 6.95–7.70 (7H, arom), 7.98 (s, H, CH), 11.53 (s, H, NH); ^13^C-NMR (ppm): 18.1, 55.3, 65.2, 101.5, 111.2, 112.3, 113.3, 114.2, 114.5, 126.3, 128.6, 128.8, 143.8, 147.9, 153.3, 154.5, 160.1, 160.8, 161.3, 163.3, 168.2; MS *m*/*z*: 365.20 [M − H]^+^, (*M*_r_ = 366.37).

*(E)-N′-(4-(Dimethylamino)benzylidene)-2-((4-methyl-2-oxo-2H-chromen-7-yl)oxy)acetohydrazide* (**29**). Yield 70%; m.p. = 262 °C; *R*_f_ = 0.4; ^1^H-NMR (ppm): 2.40 (s, 3H, CH_3_), 2.97 (s, 6H, 2(CH_3_)), 4.76–5.25 (s, 2H, CH_2_), 6.22 (s, H, CH), 6.72–7.54 (7H, arom), 7.89 (s, H, CH), 11.44 (s, H, NH); ^13^C-NMR (ppm): 18.6, 65.7, 101.9, 102.1, 111.7, 112.2, 112.8, 113.8, 121.7, 126.8, 127.0, 128.8, 145.3, 149.3, 151.9, 153.8, 155.0, 160.6, 161.9, 163.3, 168.2; MS *m*/*z*: 378.20 [M − H]^+^, (*M*_r_ = 379.41).

*(E)-N′-((3-(4-Chlorophenyl)-1-(4-fluorophenyl)-1H-pyrazol-4-yl)methylene)-2-((4-methyl-2-oxo-2H-chromen-7-yl)oxy)acetohydrazide* (**30**). Yield 75%; m.p. = 200 °C; *R*_f_ = 0.5; ^1^H-NMR (ppm): 2.40 (s, 3H, CH_3_), 4.82–5.20 (s, 2H, CH_2_), 6.22 (s, H, CH), 6.93–7.86 (12H, arom), 8.49 (s, H, CH), 11.82 (s, H, NH); ^13^C-NMR (ppm): 18.6, 65.5, 102.2, 111.7,113.9, 123.4,123.8, 126.8, 128.6, 129.3, 135.6, 142.9, 146.5, 153.8, 154.9, 160.6, 161.7, 164.3, 168.8; MS *m*/*z*: 529.30 [M + H]^+^, (*M*_r_ = 530.94).

*(E)-N′-((3-(4-Bromophenyl)-1-(p-tolyl)-1H-pyrazol-4-yl)methylene)-2-((4-methyl-2-oxo-2H-chromen-7-yl)oxy)acetohydrazide* (**31**). Yield 83%; m.p. = 248–251 °C; *R*_f_ = 0.47; ^1^H-NMR (ppm): 2.35 (s, 3H, CH_3_), 2.49 (s, 3H, CH_3_), 4.76–5.11 (s, 2H, CH_2_), 6.22 (s, H, CH), 6.88–7.87 (12H, arom), 8.97 (s, H, CH), 11.47 (s, H, NH); ^13^C-NMR (ppm): 18.1, 20.4, 65.2, 101,7, 111.4,118.4, 121.8, 126.4, 129.9, 130.4, 131.4, 131.7, 136.5, 153.3, 154.5, 160.1; MS *m*/*z*: 570.80 [M − H]^+^, (*M*_r_ = 571.43).

*(E)-N′-((3-(4-iodophenyl)-1-(p-tolyl)-1H-pyrazol-4-yl)methylene)-2-((4-methyl-2-oxo-2H-chromen-7-yl)oxy)acetohydrazide* (**32**). Yield 73%; m.p. = 265 °C; *R*_f_ = 0.45; ^1^H-NMR (ppm): 2.26 (s, 3H, CH_3_), 2.41 (s, 3H, CH_3_), 5.32 (s, 2H, CH_2_), 6.21 (s, H, CH), 6.95–7.79 (12H, arom), 8.97 (s, H, CH), 10.91 (s, H, NH); ^13^C-NMR (ppm): 18.1, 65.2, 95.8, 101.6, 111.2,112.3, 113.3, 126.3, 128.3, 129.5, 130.4, 137.5,147.8, 153.2, 154.6, 160.0, 161.4; MS *m*/*z*: 617.20 [M − H]^+^, (*M*_r_ = 618.08)

*(E)-N′-((3-(4-methoxyphenyl)-1-(p-tolyl)-1H-pyrazol-4-yl)methylene)-2-((4-methyl-2-oxo-2H-chromen-7-yl)oxy)acetohydrazide* (**33**). Yield 93%; m.p. = 238 °C; *R*_f_ = 0.41; ^1^H-NMR (ppm): 2.36 (s, 3H, CH_3_), 2.40 (s, 3H, CH3), 3.79-3.83 (s, 3H, OCH_3_), 4.78–5.18 (s, 2H, CH_2_), 6.22 (s, H, CH), 7.05-8.12 (12H, arom), 8.94 (s, H, CH), 11.48 (s, H, NH); ^13^C-NMR (ppm): 18.6, 20.9, 55.7, 101.9, 111.7,112.7, 114.4, 118.9, 126.9, 128.4, 130.2, 130.4, 136.7, 137.3, 147.8, 153.8, 168.3; MS *m*/*z*: 521.40 [M − H]^+^, (*M*_r_ = 522.2).

*(E)-N′-((3-(4-Chlorophenyl)-1-(p-tolyl)-1H-pyrazol-4-yl)methylene)-2-((4-methyl-2-oxo-2H-chromen-7-yl)oxy)acetohydrazide* (**34**). Yield 65%; m.p. = 255 °C; *R*_f_ = 0.47; ^1^H-NMR (ppm): 2.36 (s, 3H, CH_3_), 2.40 (s, 3H, CH_3_), 4.79–5.13 (s, 2H, CH_2_), 6.23 (s, H, CH), 6.90–8.12 (12H, arom), 8.98 (s, H, CH), 11.50 (s, H, NH); ^13^C-NMR (ppm): 18.6, 20.9, 101.9, 102.2, 111.7, 112.7, 119.1, 119.3, 126.9, 129.0, 129.2, 130.5, 130.6, 153.8; MS *m*/*z*: 525.30 [M − H]^+^, (*M*_r_ = 526.98).

*(E)-N′-((1-(4-Chlorophenyl)-3-(4-nitrophenyl)-1H-pyrazol-4-yl)methylene)-2-((4-methyl-2-oxo-2H-chromen-7-yl)oxy)acetohydrazide* (**35**). Yield 69%; m.p. = 281 °C; *R*_f_ = 0.42; ^1^H-NMR (ppm): 2.40 (s, 3H, CH_3_), 4.81–5.09 (s, 2H, CH_2_), 6.23 (s, H, CH), 6.86–8.47 (12H, arom), 9.11 (s, H, CH), 11.59 (s, H, NH); ^13^C-NMR (ppm): 18.6, 101.9, 111.8, 112.6, 121.5, 124.1, 124.2, 126.8, 130.0, 130.2, 168.4; MS *m*/*z*: 556.30 [M − H]^+^, (*M*_r_ = 557.94).

*(E)-N′-((4-Chloro-2-oxo-2H-chromen-3-yl)methylene)-2-((4-methyl-2-oxo-2H-chromen-7-yl)oxy)acetohydrazide* (**36**). Yield 78%; m.p. = 233 °C; *R*_f_ = 0.28; ^1^H-NMR (ppm): 2.40 (s, 3H, CH_3_), 4.78–5.25 (s, 2H, CH_2_), 6.21 (s, H, CH), 6.91–8.03 (12H, arom), 8.21 (s, H, CH), 11.98 (s, H, NH); ^13^C-NMR (ppm): 18.1, 65.2, 101.4, 111.3, 112.1, 113.4, 116.5, 118.4, 125.3, 126.1, 126.4, 133.6, 1370.5, 153.3, 154.5; MS *m*/*z*: 439.20 [M + H]^+^, (*M*_r_ = 438.82).

### 3.5. Determination of DPPH Scavenging Activity

DPPH scavenging activity for previously synthesized compounds [2,5] and new compounds (**30**–**36**) was performed according to the procedure described in our previous work [4]. For each sample, antioxidant activity determination was performed in triplicate and expressed as % scavenging activity (% DPPH).

### 3.6. QSAR Studies

#### 3.6.1. Data Set

The dataset used for building QSAR models consists of 36 molecules whose antioxidant activities were measured and described in the present study. Antioxidant activity, expressed as % DPPH), were converted in the form of the logarithm (log % DPPH) and presented in Table 1 together with structures of molecules (While transformation of the experimental data to both logit % DPPH and log %DPPH afforded a normal distribution, the latter approach afforded a QSAR model, which appears to provide a better relationship between the structures of the molecules and their activities).

#### 3.6.2. Descriptor Calculation and Selection

The 3D structures of 36 molecules were optimized applying the Avogadro 1.2.0. (University of Pittsburgh, Pittsburgh, PA, USA) [46] using the molecular mechanics force field (MM+) [47]. Subsequently, all structures were submitted to geometry optimization using the semiempirical AM1 method [48]. Two sets of descriptors were generated using Parameter Client (Virtual Computational Chemistry Laboratory, an electronic remote version of the Dragon program [49]). In order to reduce the huge number of calculated descriptors (about 1260), firstly, zero value descriptors were excluded from the initial pool. Further exclusion was performed using QSARINS-Chem 2.2.1 (University of Insubria, Varese, Italy) [50]: constant and semi-constant descriptors, i.e., those having chemical compounds with a constant value for more than 80%, and descriptors that are too inter-correlated (>85%) were rejected. Data sets were randomly divided into training (80%, *N*train = 29) and test (20%, *N*test = 7) set using QSARINS.

#### 3.6.3. Regression Analysis and Validation of Models

The best QSAR models were obtained by Genetic Algorithm (GA) using QSARINS. The number of descriptors (*I*) in the multiple regression equation was limited to three. The models have been assessed by: fitting criteria; internal cross-validation using the leave-one out (LOO) method and Y-scrambling; and external validation. Fitting criteria included: the coefficient of determination (*R*^2^), adjusted (*R*^2^_adj_), cross-validate *R*^2^ using the leave-one-out method (*Q*^2^_LOO_), global correlation among descriptors (*Kxx*), the difference between global correlation between molecular descriptors and *y* the response variable, and global correlation among descriptors (Δ*K*), standard deviation of regression (*s*), and Fisher ratio (*F*) [32,51,52]. Internal and external validations also included the following parameters: root-mean-square error of the training set (*RMSE_tr_*); root-mean-square error of the training set determined through the cross validated LOO method (*RMSE_cv_*), root-mean-square error of the external validation set (*RMSE_ex_*), squared correlation coefficients between the observed and (leave-one-out) predicted values of the compounds with and without intercept ($r_{m\;}^{2}$); concordance correlation coefficient of the training set (*CCC_tr_*), test set using LOO cross validation (*CCC_cv_*), and of the external validation set (*CCC_ex_*), mean absolute error of the training set (*MAE_tr_*), mean absolute error of the internal validation set (*MAE_cv_*) and mean absolute error of the external validation set (*MAE_ex_*) predictive residual sum of squares determined through cross-validated LOO method (*PRESS_cv_*) in the training set and in the external prediction set (*PRESS_ex_*) [23,51]. The analysed external validation parameters also include the coefficient of determination (*R*^2^*_ex_*). Robustness of QSAR models was tested by a Y-randomisation test.

Investigation of the applicability domain of a prediction model was performed by leverage plot (plotting residuals vs. leverage of training compounds). The warning leverage h* is defined as 3*p*′/*n*, where *n* is the number of training compounds and *p*′ is the number of model adjustable parameters [37]. Tools of regression diagnostics as residual plots and Williams plots were used to check the quality of the best models and define their applicability domain using QSARINS.

#### 3.6.4. Visualization of Electrostatic Potential Surface

Electrostatic potential surface has been generated from optimized structures by ArgusLab 4.0.1 (Mark A. Thompson, Planaria Software LLC, Seattle, WA, USA). In an ESP-mapped density surface, the electron density surface gives the shape of the surface, while the value of the ESP on that surface gives the colors. Maximum mapped surface was set to 0.05, while the minimum to −0.05 a.u.

## 4. Conclusions

A series of novel coumarinyl Schiff bases in DES have been synthesized and evaluated for antioxidant activity. Compared to previously synthesized coumarin derivatives, novel compounds have not showed an improvement in antioxidant effect. QSAR study has clarified the importance of two hydroxyl groups in ortho position on phenyl ring and hydrophilicity for enhanced antioxidant activity coumarin derivatives. Electrostatic potential surface provides a visualization of possible regions in molecules that allow easy electron transfer to DPPH radicals.

## Figures and Tables

**Table:** 

Compound	R	Compound	R	Compound	R
**4**	H	**17**	3-O-Ph	**30**	R_1_ = Cl; R_2_ = F
**5**	2-OH	**18**	2-OH; 5-NO_2_	**31**	R_1_ = Br; R_2_ = CH_3_
**6**	3-OH	**19**	3,4,5-OCH_3_	**32**	R_1_ = I; R_2_ = CH_3_
**7**	4-OH	**20**	2-Cl	**33**	R_1_ = OCH_3_; R_2_ = CH_3_
**8**	2-OCH_3_	**21**	3-Cl	**34**	R_1_ = Cl; R_2_ = CH_3_
**9**	3-OCH_3_	**22**	2-Br	**35**	R_1_ = NO_2_; R_2_ = Cl
**10**	4-OCH_3_	**23**	3-Br		
**11**	2,3-OH	**24**	4-Br		
**12**	2,4-OH	**25**	2-F		
**13**	2,5-OH	**26**	3-F		
**14**	3,4-OH	**27**	4-F		
**15**	3,5-OH	**28**	styryl		
**16**	3-OCH_3_; 4-OH	**29**	4-N(CH_3_)_2_		

**Table 1:** Structures of analysed compounds with their antioxidant activities (%DPPH) *.

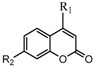			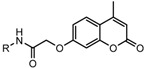		
**Mol. ID**	**R_1_/R_2_**	**% DPPH**	**Mol. ID**	**R**	**% DPPH**
**1**	H-/H-	1.8 ± 0.22	**3**	H_2_N-	14.4 ± 0.81
**2**	-CH_3_/-OH	2.4 ± 0.12	**28**	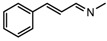	1.6 ± 0.00
			**36**	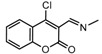	14.1 ± 0.10
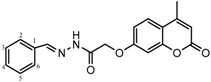					
**Mol. ID**	**Supstituents**	**% DPPH**	**Mol. ID**	**Supstituents**	**% DPPH**
**4**	H	22.9 ± 0.50	**16**	3-OCH_3_; 4-OH	32.0 ± 0.25
**5**	2-OH	16.6 ± 0.56	**17**	3-O-Ph	2.2 ± 0.68
**6**	3-OH	3.6 ± 0.24	**18**	2-OH; 5-NO_2_	2.9 ± 0.99
**7**	4-OH	4.4 ± 0.37	**19**	3,4,5-OCH_3_	3.5 ± 0.42
**8**	2-OCH_3_	3.1 ± 0.86	**20**	2-Cl	11.1 ± 0.87
**9**	3-OCH_3_	0.2 ± 0.08	**21**	3-Cl	7.5 ± 0.37
**10**	4-OCH_3_	3.7 ± 0.23	**22**	2-Br	6.6 ± 0.62
**11**	2,3-OH	75.4 ± 2.62	**23**	3-Br	3.0 ± 0.32
**12**	2,4-OH	8.4 ± 0.12	**24**	4-Br	5.0 ± 0.27
**13**	2,5-OH	33.4 ± 3.74	**25**	2-F	3.0 ± 0.76
**14**	3,4-OH	42.6 ± 4.10	**26**	3-F	2.3 ± 0.68
**15**	3,5-OH	4.6 ± 2.17	**27**	4-F	7.4 ± 0.12
			**29**	4-N(CH_3_)_2_	5.9 ± 0.62
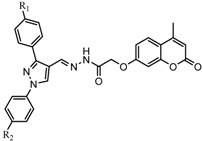					
**Mol. ID**	**R**	**% DPPH**	**Mol. ID**	**R**	**% DPPH**
**30**	R_1_ = Cl; R_2_ = F	1.6 ± 0.23	**33**	R_1_ = OCH_3_; R_2_ = CH_3_	0.8 ± 0.09
**31**	R_1_ = Br; R_2_ = CH_3_	0.5 ± 0.01	**34**	R_1_ = Cl; R_2_ = CH_3_	1.4 ± 0.19
**32**	R_1_ = I; R_2_ = CH_3_	1.6 ± 0.18	**35**	R_1_ = NO_2_; R_2_ = Cl	1.6 ± 0.31

* data are means ± standard deviation of three replicates.

**Table 2:** The model statistical results for the QSAR for antioxidant activity.

	Model (1)	Model (2)
**Fittinig criteria**		
*R*^2^	0.636	0.673
*R*^2^_adj_	0.592	0.632
*s*	0.369	0.315
*F*	14.559	16.467
*Kxx*	0.242	0.241
*ΔK*	0.170	0.178
*RMSE_tr_*	0.342	0.292
*MAE_tr_*	0.271	0.241
*CCC_tr_*	0.778	0.805
**Internal validation criteria**		
*Q*^2^*_LOO_*	0.512	0.544
*RMSE_cv_*	0.397	0.345
*MAE_cv_*	0.314	0.285
*PRESS_cv_*	4.560	3.326
*CCC_cv_*	0.710	0.733
*R*^2^*_Y_* *scr*	0.108	0.112
*Q*^2^*_Y_* *scr*	−0.214	−0.221
**External validation criteria**		
*RMSE_ext_*	0.299	0.311
*MAE_ext_*	0.271	0.283
*PRESS_ext_*	0.627	0.677
*R*^2^*_ext_*	0.709	0.712
*Q*^2^*_F_*_1_	0.523	0.558
*Q*^2^*_F_*_2_	0.169	0.103
*Q*^2^*_F_*_3_	0.722	0.629
*CCC_ext_*	0.732	0.701
$\bar{r_{m}^{2}}$	0.619	0.600
$\Delta r_{m}^{2}$	0.050	0.197
Applicability domain (h* = 0.4138)		
*N* compounds outlier	1 (**9**)	0
*N* compounds out of app.dom.	0	0

LOO (the leave-one out); *R*^2^ (coefficient of determination); *R*^2^_adj_ (adjusted coefficient of determination); *s* (standard deviation of regression); *F* (Fisher ratio); *Kxx* (global correlation among descriptors); *ΔK* (global correlation among descriptors); *RMSE_tr_* (root-mean-square error of the training set); *MAE_tr_* (mean absolute error of the training set); *CCC_tr_* (concordance correlation coefficient of the training set); *Q^2^_LOO_* (cross-validated explained variance); *RMSE_cv_* (root-mean-square error of the training set determined through the cross validated method; *MAE_cv_* (mean absolute error of the internal validation set); *PRESS_cv_* (predictive residual sum of squares determined through cross-validated method); *CCC_cv_* (concordance correlation coefficient test set using cross validation); *R*^2^*_Y_scr* (Y-scramble correlation coefficients); *Q*^2^*_Yscr_* (Y-scramble cross-validation coefficients); *RMSE_ex_* (root-mean-square error of the external validation set); *MAE_ex_* (mean absolute error of the external validation set); *PRESS_ext_* (predictive residual sum of squares determined through cross-validated LOO method in the external prediction set; *R*^2^*_ext_* (coefficient of determination of validation set); *Q*^2^*_F_*_1_, *Q*^2^*_F_*_2_, *Q*^2^*_F_*_3_ (predictive squared correlation coefficients); *CCC_ext_* (concordance correlation coefficient of the test set); $\bar{r_{m}^{2}}$ (average value of squared correlation coefficients between the observed and (leave-one-out) predicted values of the compounds with and without intercept); $\Delta r_{m}^{2}$ (absolute difference between the observed and leave-one-out predicted values of the compounds with and without intercept); h* (warning leverage for the applicability domain of the model).

**Table 3:** Correlation matrix for the descriptors included in model 1.

	*MATS3m*	*Mor22u*	*Hy*
*MATS3m*	1		
*Mor22u*	−0.260	1	
*Hy*	0.194	0.309	1

## Supplementary Materials

Supplementary materials are available online. File S1: ^1^H- and ^13^C-NMR spectra of synthesized compounds; Table S2: Values of the descriptors included in models (1–2), as experimental and calculated log %DPPH.
