# Supplementary material for: Deep Eutectic Solvents as Convenient Media for Synthesis of Novel Coumarinyl Schiff Bases and Their QSAR Studies
Source: Molecules. 2017 Sep 5;22(9):1482. doi: 10.3390/molecules22091482 (PMC6151826; doi:10.3390/molecules22091482)
Supplement: Supplementary file 1 [file molecules-22-01482-s001.zip › Molnar Table S2.pdf]

Table S2: Values of the descriptors included in models (1-2), as experimental and calculated log %DPPH.

Mol. ID	*Mor22u*	*MATS3m*	*Hy*	Status	Exp. log % DPPH	Calc. log % DPPH by eq. 1	Calc. log % DPPH by eq. 1
**1**	0.252	-0.214	-0.790	Training	0.255	0.417	0.463
**2**	0.111	-0.179	-0.202	Training	0.380	0.603	0.645
**3**	0.028	0.119	1.251	Training	1.158	1.091	1.123
**4**	0.285	0.123	-0.317	Training	1.360	0.888	0.899
**5**	0.268	0.017	0.289	Training	1.220	1.118	1.122
**6***	0.267	0.048	0.289	Test	0.556	0.988	1.006
**7**	0.267	0.048	0.289	Training	0.644	0.988	1.006
**8**	0.157	0.032	-0.309	Training	0.491	0.698	0.727
**9**	-0.041	0.062	-0.309	Training	-0.699	0.274	
**10**	-0.042	0.062	-0.309	Training	0.568	0.273	0.345
**11**	0.300	0.044	0.981	Training	1.877	1.817	1.768
**12***	0.216	-0.034	0.981	Test	0.924	1.198	1.213
**13**	0.319	-0.034	0.981	Training	1.524	1.350	1.350
**14**	0.226	0.073	0.981	Training	1.629	1.613	1.584
**15**	0.146	-0.007	0.981	Training	0.663	1.000	1.035
**16**	-0.117	0.088	0.282	Training	1.505	0.809	0.840
**17**	0.082	0.039	-0.372	Training	0.342	-0.129	-0.015
**18**	-0.119	-0.084	0.330	Training	0.462	0.920	0.942
**19***	-0.274	0.125	-0.297	Test	0.544	0.600	0.635
**20**	0.055	-0.091	-0.294	Training	1.045	0.636	0.671
**21**	0.153	-0.047	-0.294	Training	0.875	0.636	0.672
**22**	0.015	-0.064	-0.294	Training	0.820	0.575	0.616
**23**	0.202	-0.031	-0.294	Training	0.477	0.807	0.826
**24***	0.217	-0.031	-0.294	Test	0.699	0.829	0.846
**25**	0.326	-0.051	-0.294	Training	0.477	0.922	0.930
**26**	0.171	-0.006	-0.294	Training	0.362	0.477	0.530
**27**	0.226	-0.006	-0.294	Training	0.869	0.558	0.603
**28***	0.170	0.157	-0.346	Test	0.204	0.605	0.643

**29**	-0.171	0.118	-0.323	Training	0.771	0.615	0.649
**30**	-0.139	-0.043	-0.348	Training	0.204	0.134	0.218
**31**	-0.346	-0.019	-0.374	Training	-0.301	-0.006	0.089
**32**	-0.277	-0.015	-0.374	Training	0.204	0.110	0.194
**33***	-0.187	0.038	-0.383	Test	-0.097	0.169	0.248
**34***	-0.427	-0.030	-0.374	Test	0.146	-0.191	-0.077
**35**	-0.148	-0.044	-0.317	Training	0.204	0.237	0.311
**36**	0.302	-0.131	-0.297	Training	1.149	1.106	1.094

* members of the test set
