# Supplementary material for: Deep Eutectic Solvents as Convenient Media for Synthesis of Novel Coumarinyl Schiff Bases and Their QSAR Studies
Source: Molecules. 2017 Sep 5;22(9):1482. doi: 10.3390/molecules22091482 (PMC6151826; doi:10.3390/molecules22091482)

Supplementary material: ^{1}H and ^{13}C NMR spectra of synthesized compounds

For manuscript entitled:

Deep eutectic solvents as convenient media for synthesis of novel coumarinyl Schiff bases and their QSAR studies

Maja Molnar [a], Mario Komar [a], Harshad Brahmbhatt[a], Jurislav Babić[a], Stela Jokić[a], Vesna Rastija[b,*]

[a]Josip Juraj Strossmayer University of Osijek, Faculty of Food Technology Osijek, Franje Kuhaca 20, 31000 Osijek, Croatia

[b]Josip Juraj Strossmayer University of Osijek, Faculty of Agriculture in Osijek, Vladimira Preloga 1, 31000 Osijek, Croatia

(E)-N′-(4-methoxybenzylidene)-2-((4-methyl-2-oxo-2H-chromen-7-yl)oxy)acetohydrazide (**10**) [2]

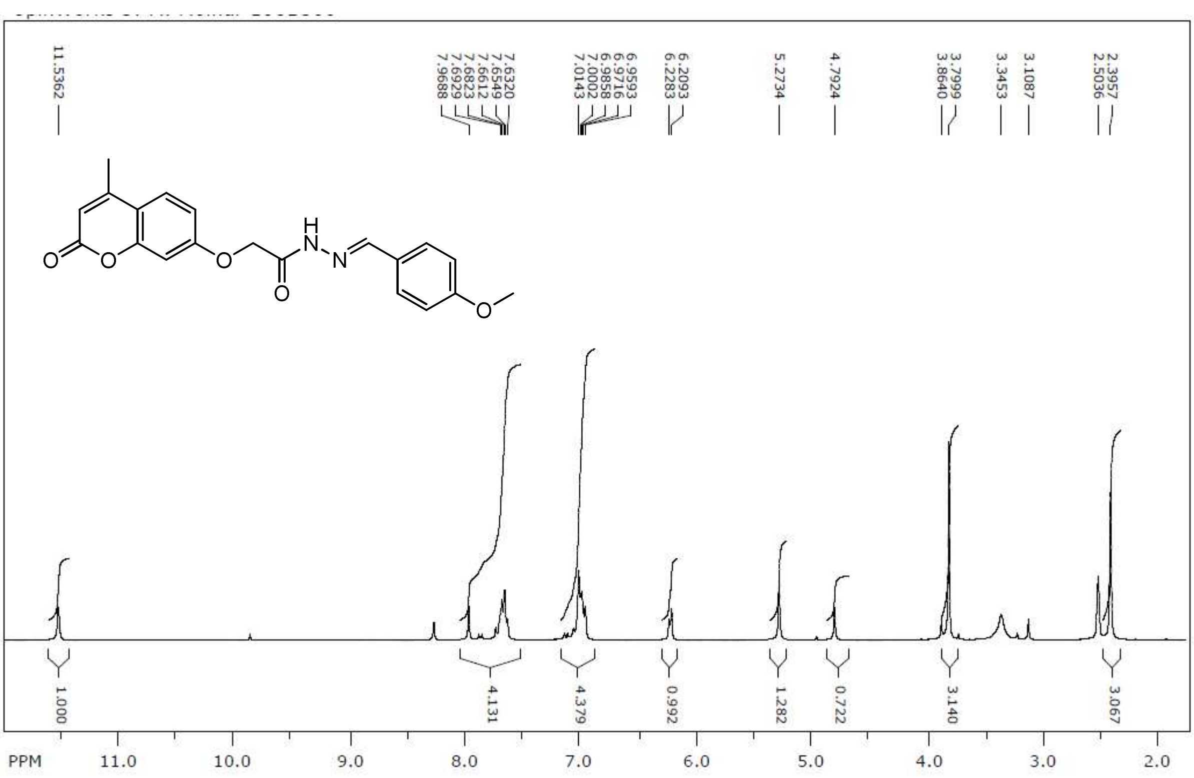

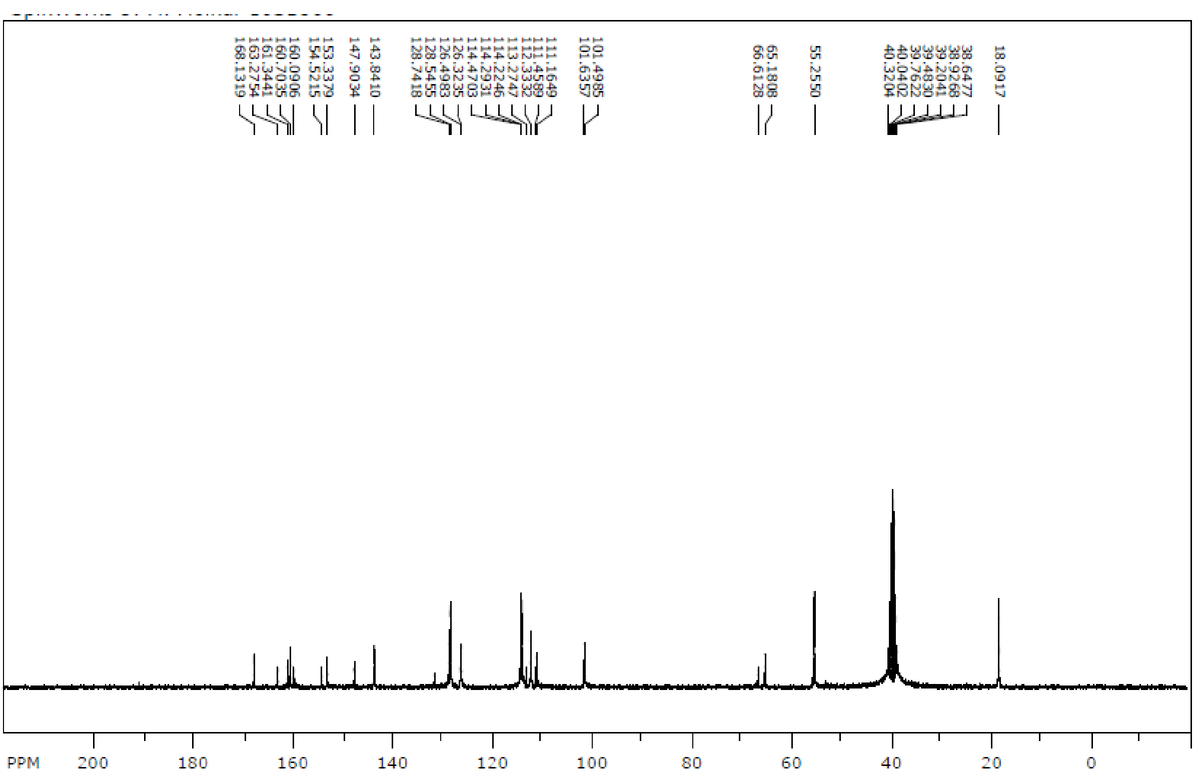

(E)-N′-(4-(dimethylamino)benzylidene)-2-((4-methyl-2-oxo-2H-chromen-7-yl)oxy)acetohydrazide (**29**) [5]

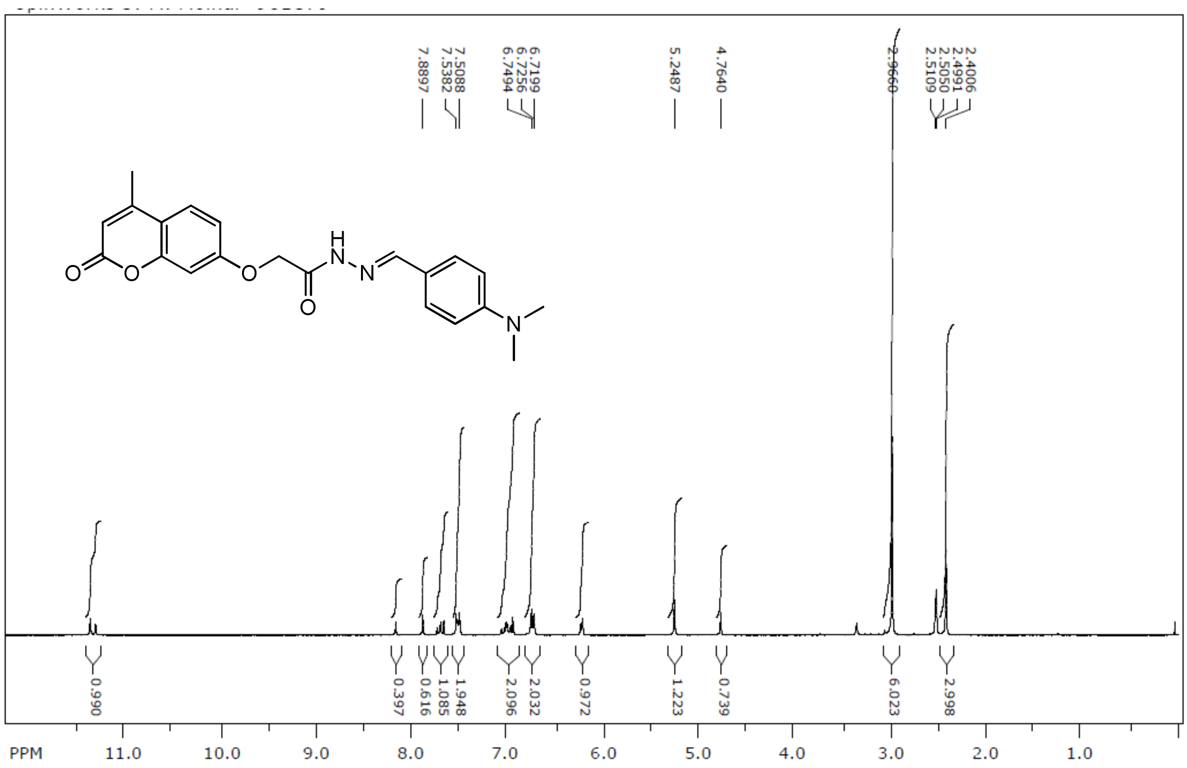

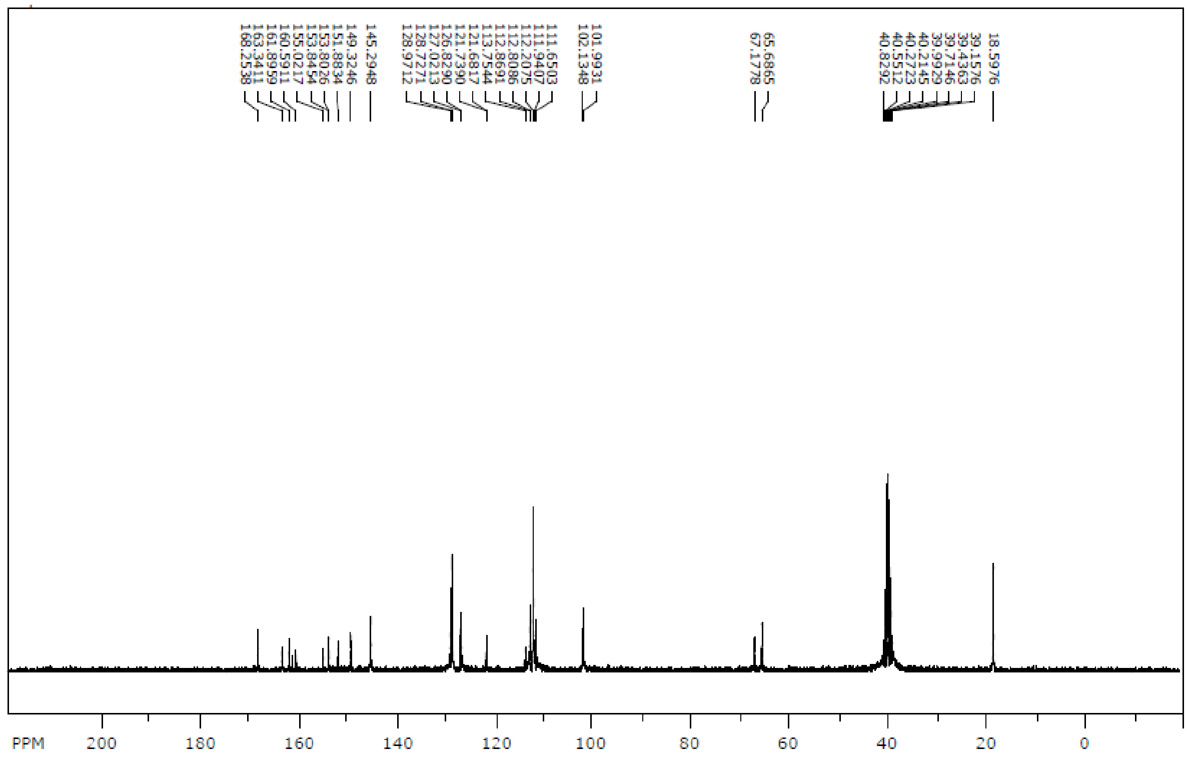

(E)-N′-((3-(4-chlorophenyl)-1-(4-fluorophenyl)-1H-pyrazol-4-yl)methylene)-2-((4-methyl-2-oxo-2H-chromen-7-yl)oxy)acetohydrazide (**30**)

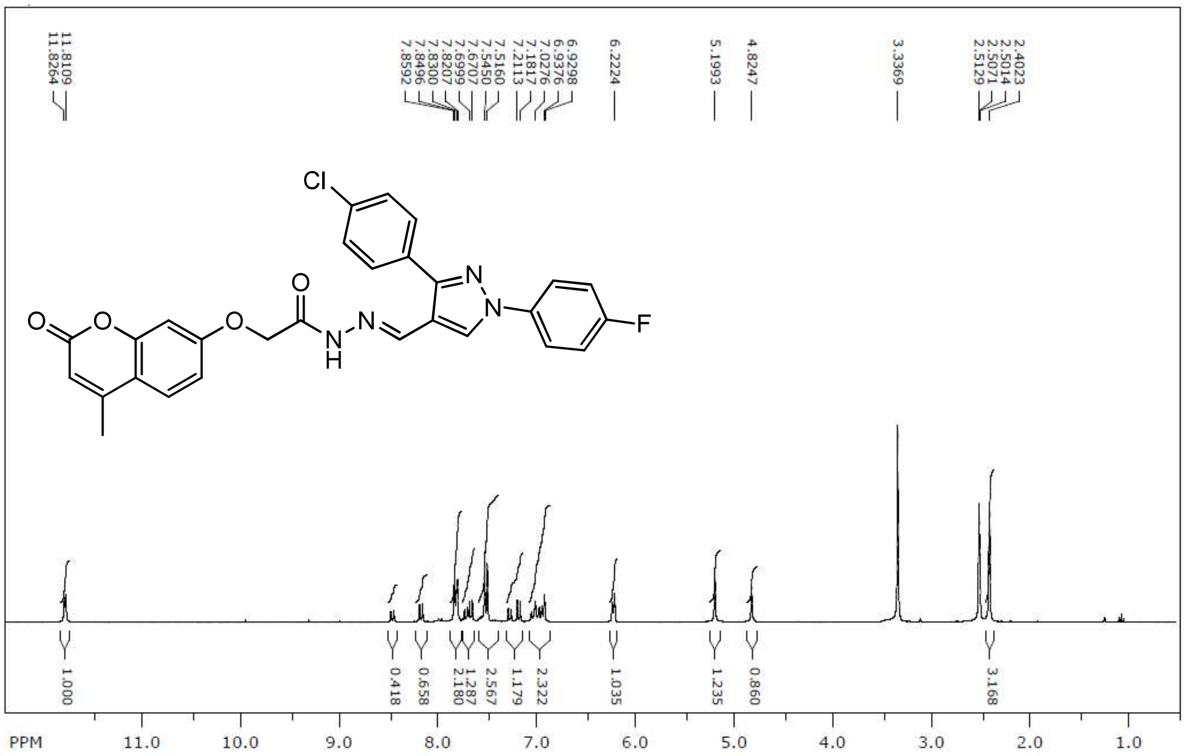

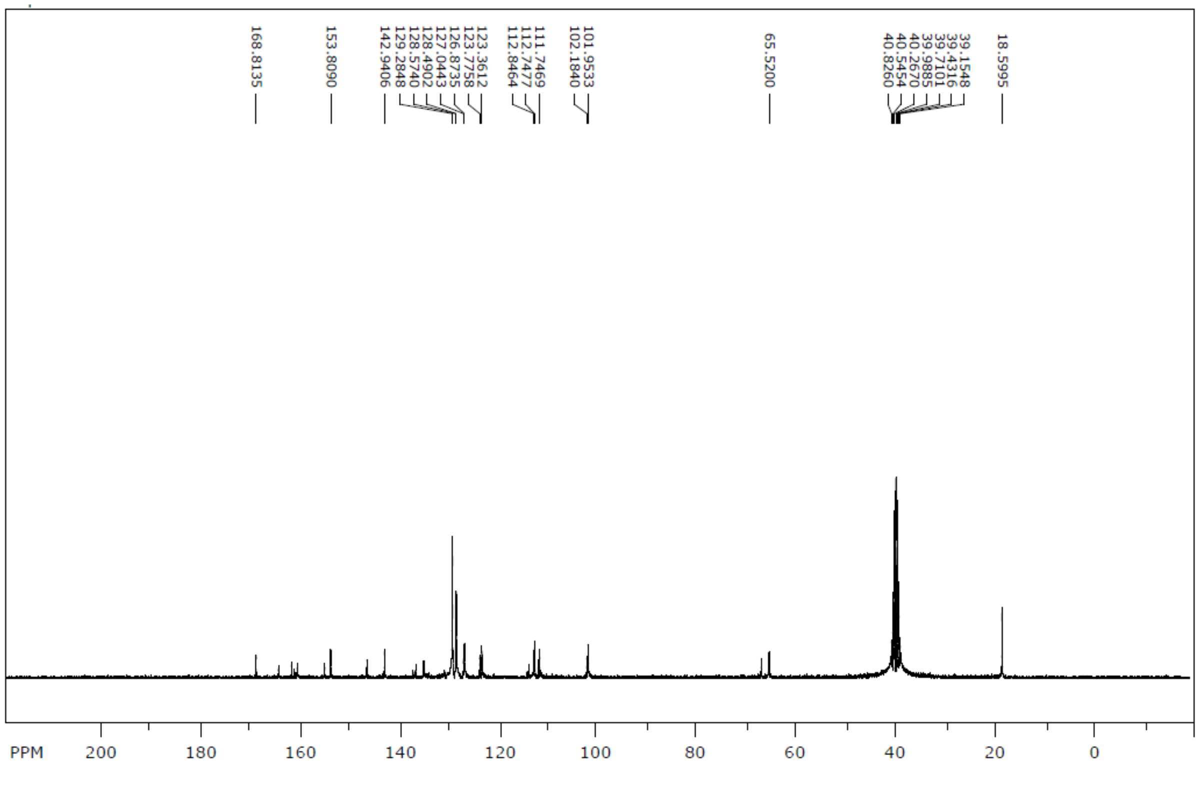

(E)-N'-((3-(4-bromophenyl)-1-(p-tolyl)-1H-pyrazol-4-yl)methylene)-2-((4-methyl-2-oxo-2H-chromen-7-yl)oxy)acetohydrazide (**31**)

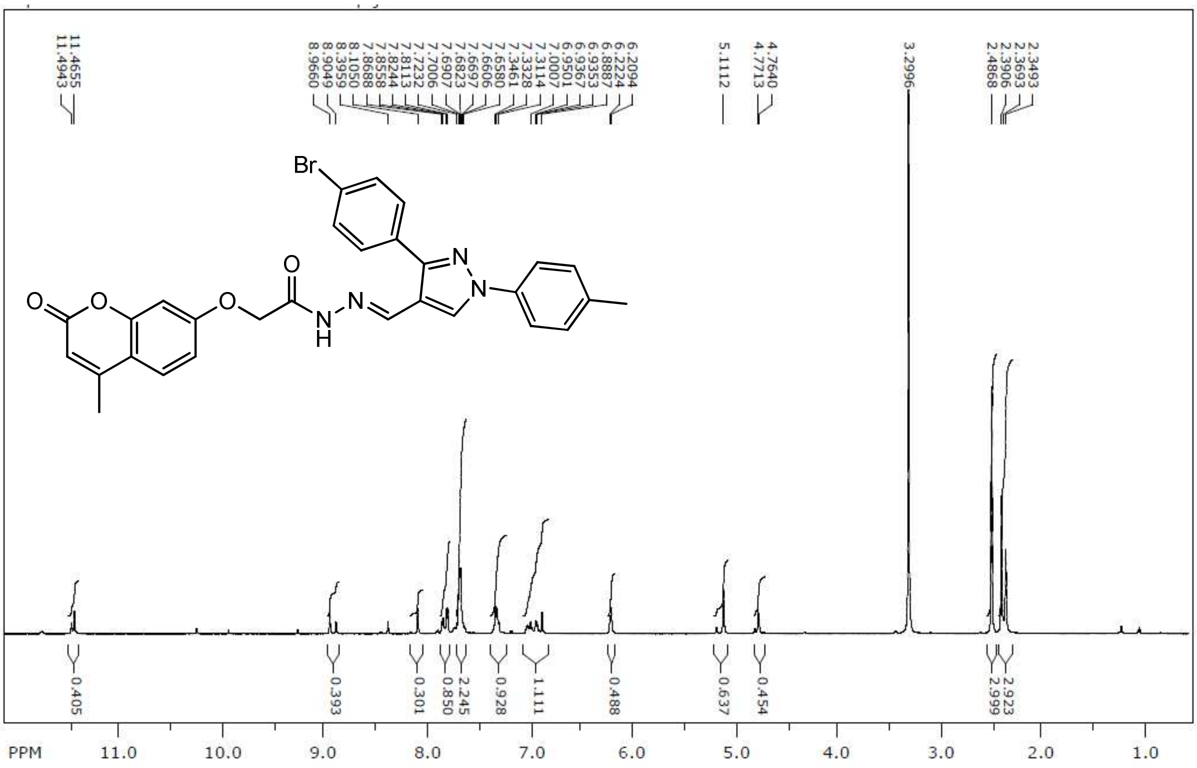

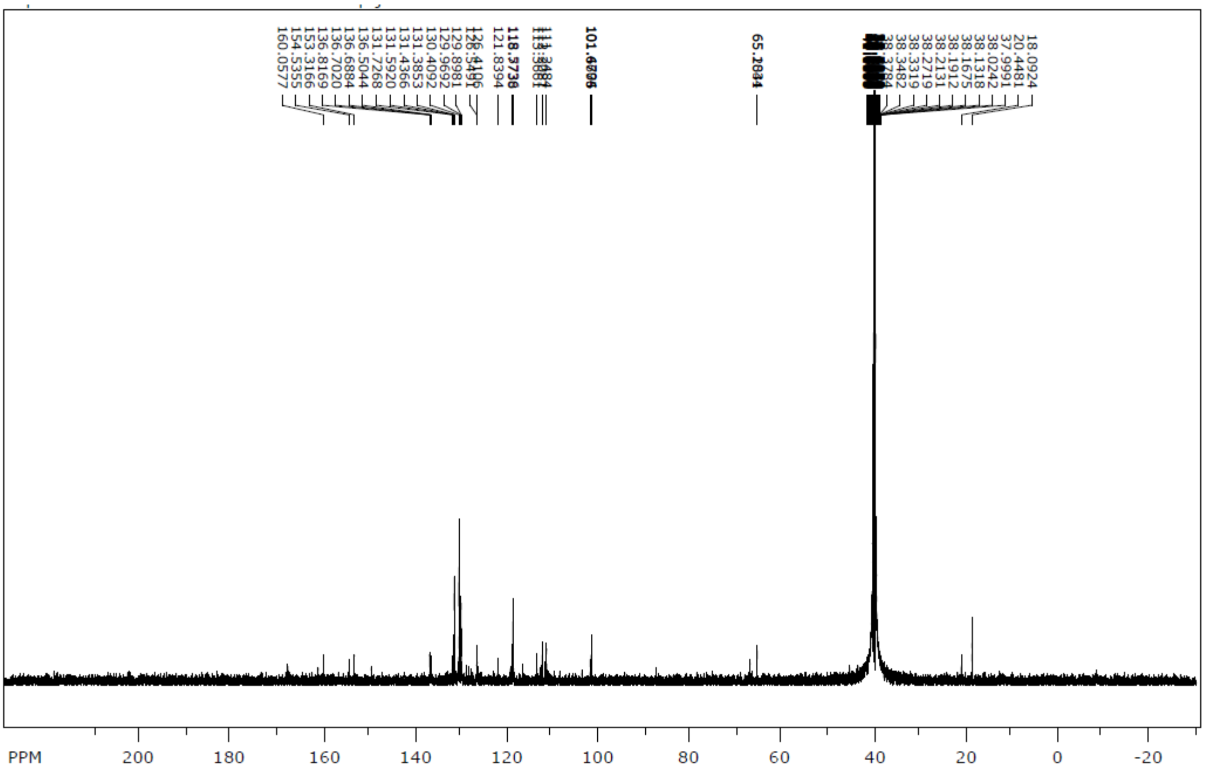

(E)-N′-((3-(4-iodophenyl)-1-(p-tolyl)-1H-pyrazol-4-yl)methylene)-2-((4-methyl-2-oxo-2H-chromen-7-yl)oxy)acetohydrazide (**32**)

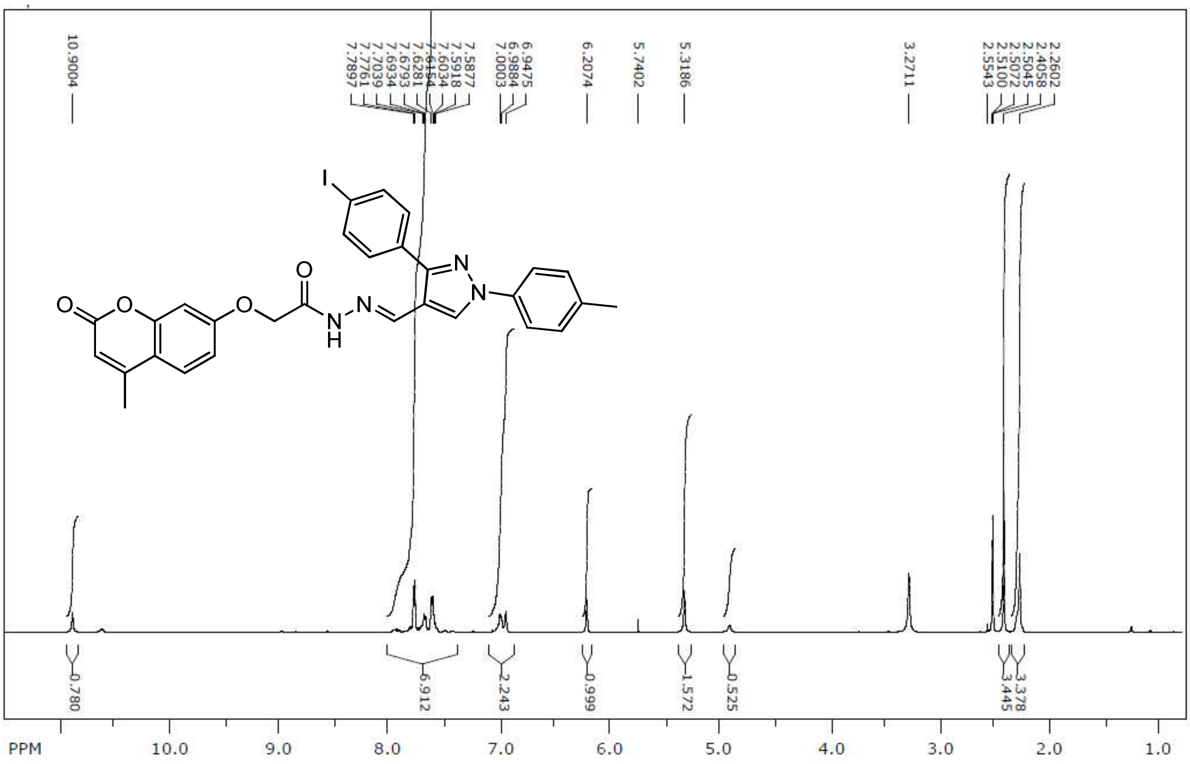

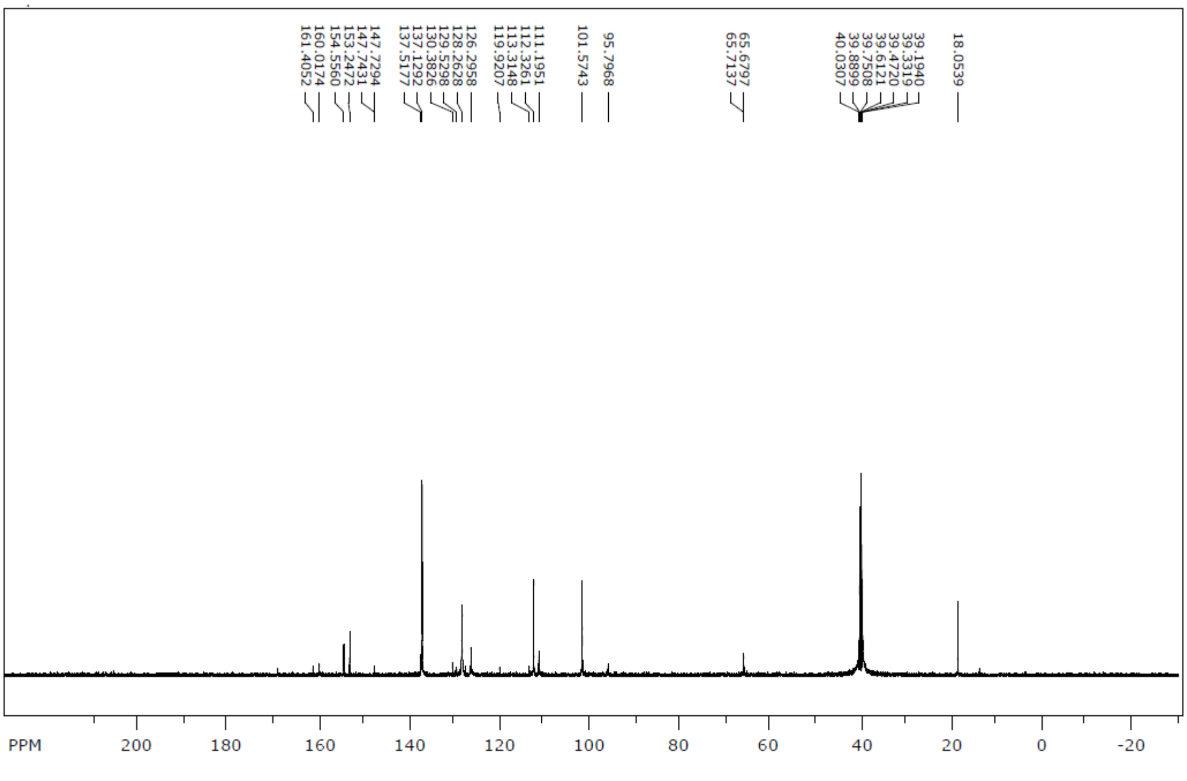

(E)-N′-((3-(4-methoxyphenyl)-1-(p-tolyl)-1H-pyrazol-4-yl)methylene)-2-((4-methyl-2-oxo-2H-chromen-7-yl)oxy)acetoh ydrazide (**33**)

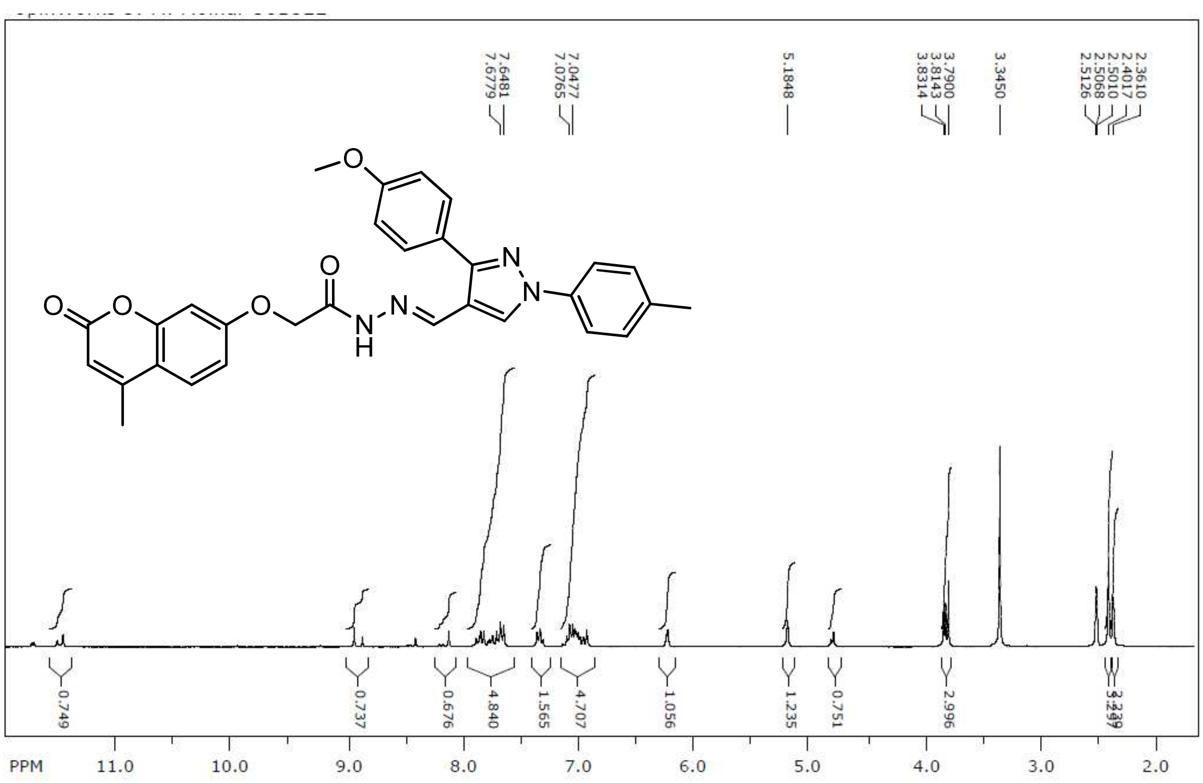

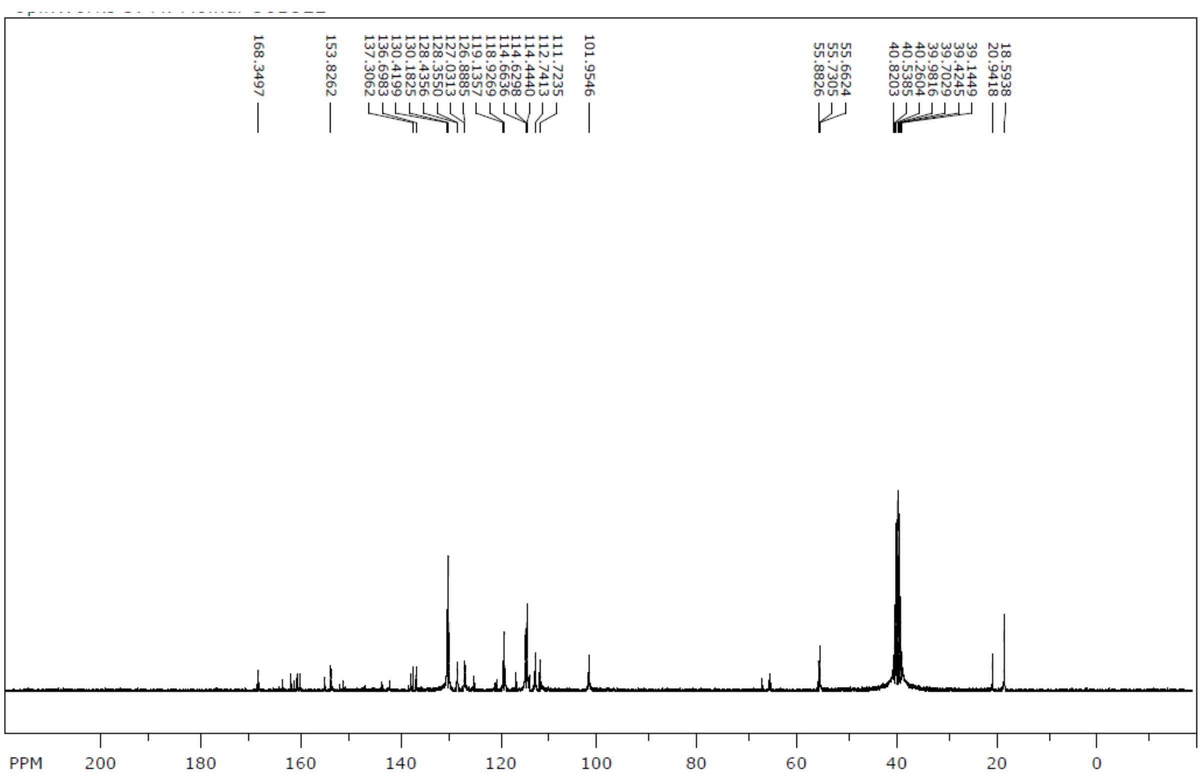

(E)-N'-((3-(4-chlorophenyl)-1-(p-tolyl)-1H-pyrazol-4-yl)methylene)-2-((4-methyl-2-oxo-2H-chromen-7-yl)oxy)acetohydrazide (**34**)

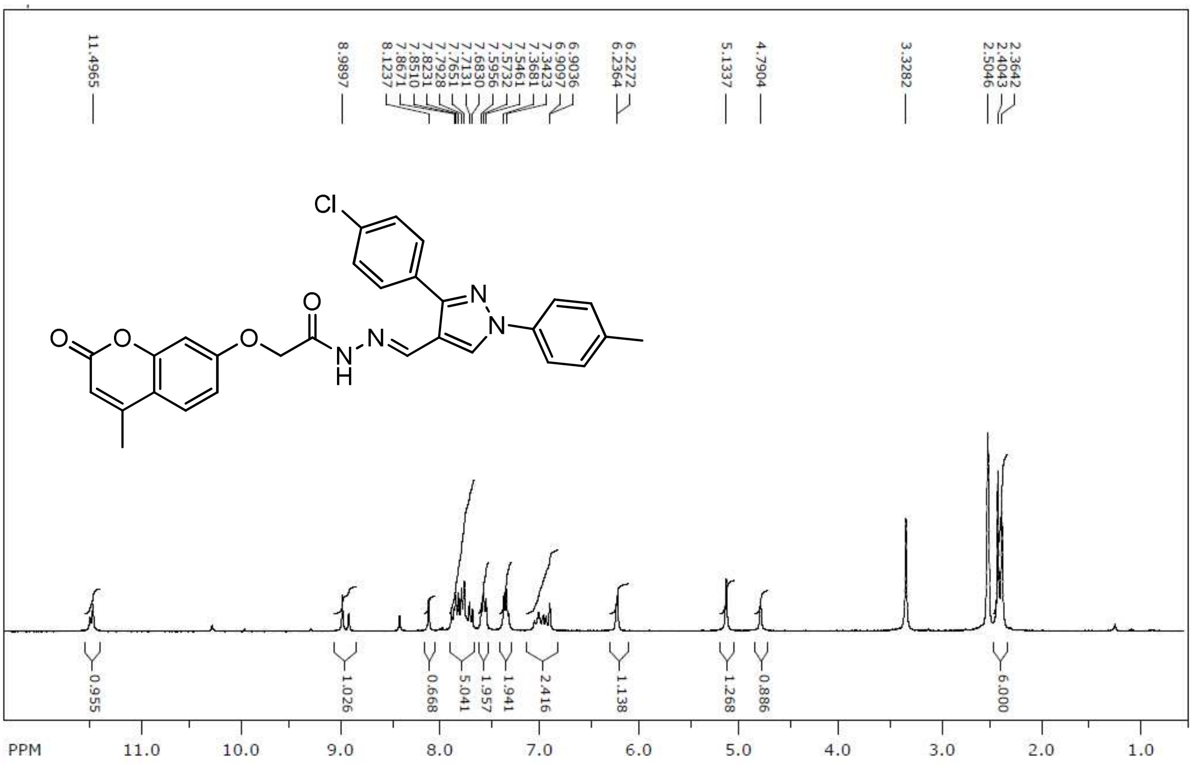

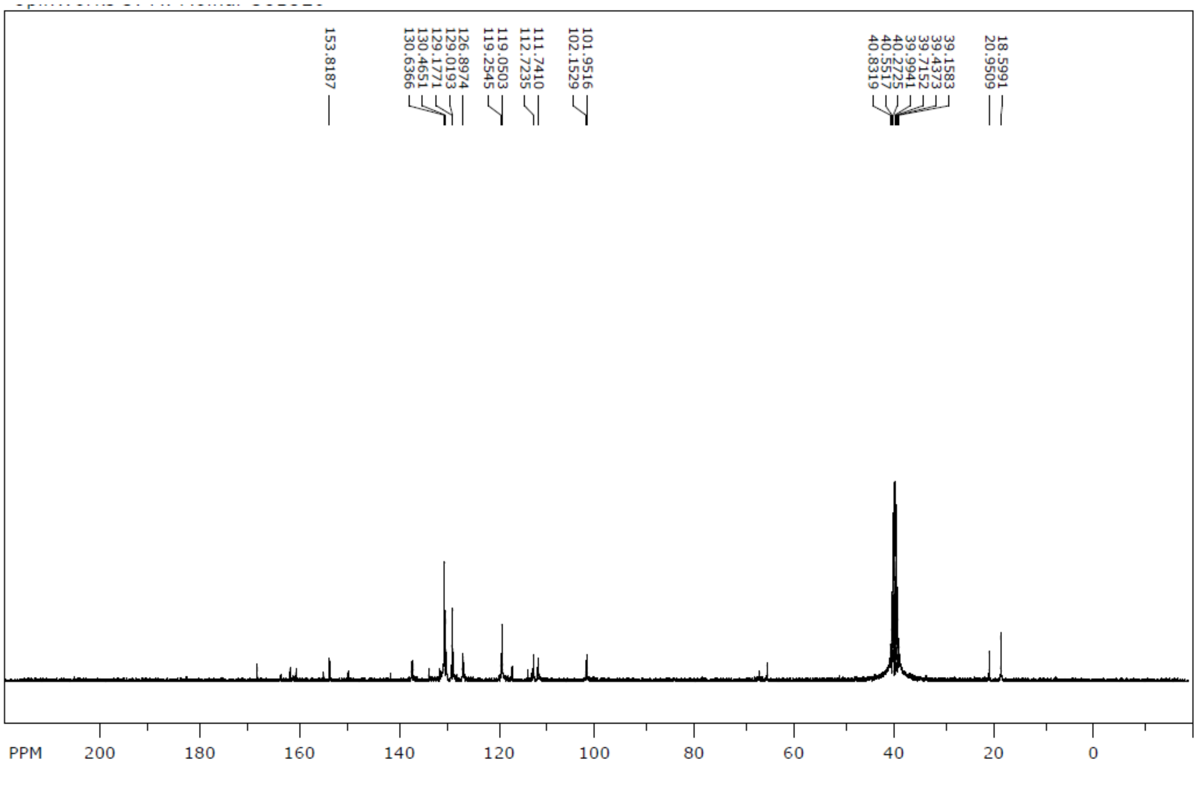

(E)-N′-((1-(4-chlorophenyl)-3-(4-nitrophenyl)-1H-pyrazol-4-yl)methylene)-2-((4-methyl-2-oxo-2H-chromen-7-yl)oxy)acetohydrazide (**35**)

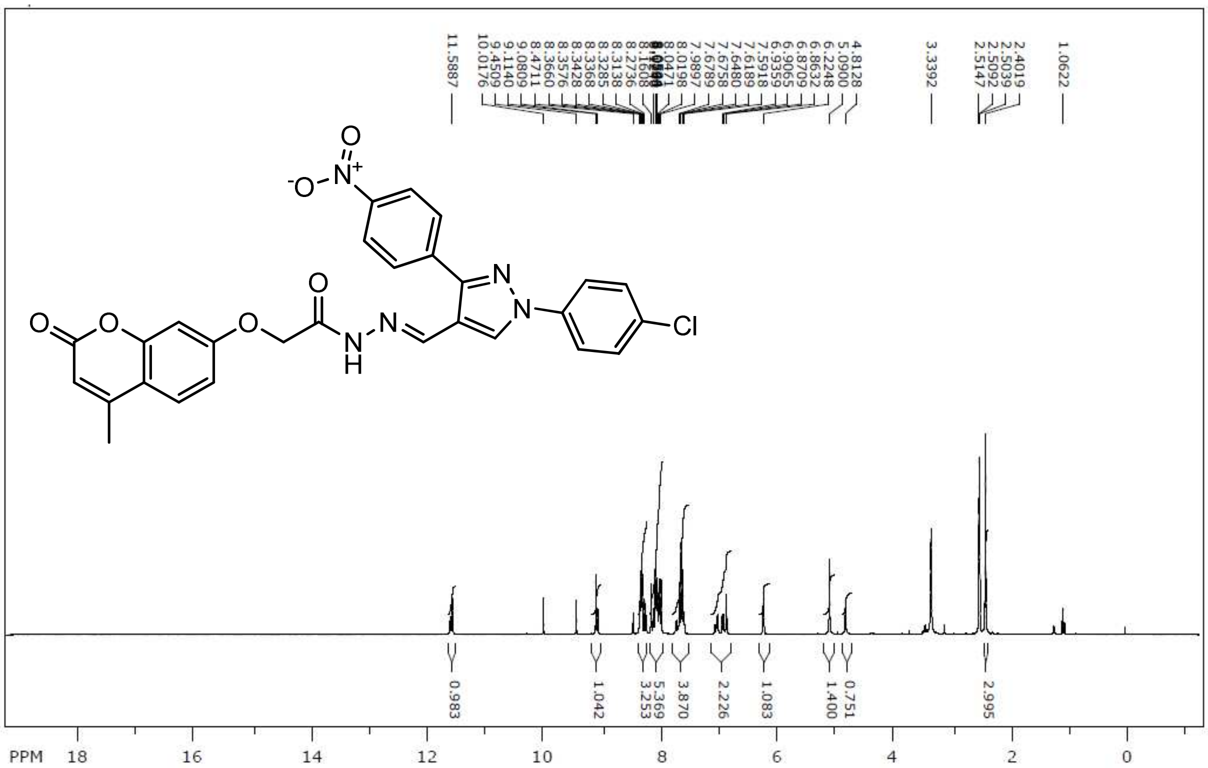

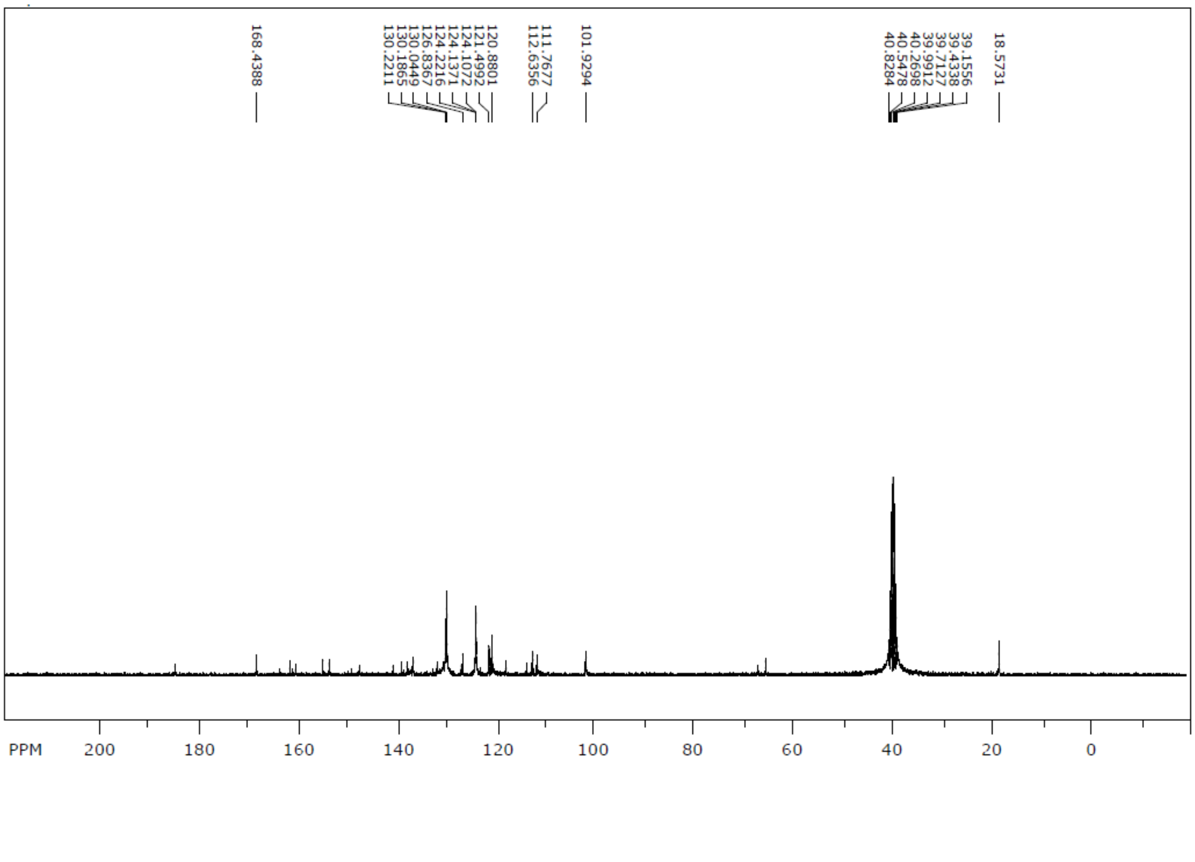

(E)-N′-((4-chloro-2-oxo-2H-chromen-3-yl)methylene)-2-((4-methyl-2-oxo-2H-chromen-7-yl)oxy)acetohydrazide (**36**)

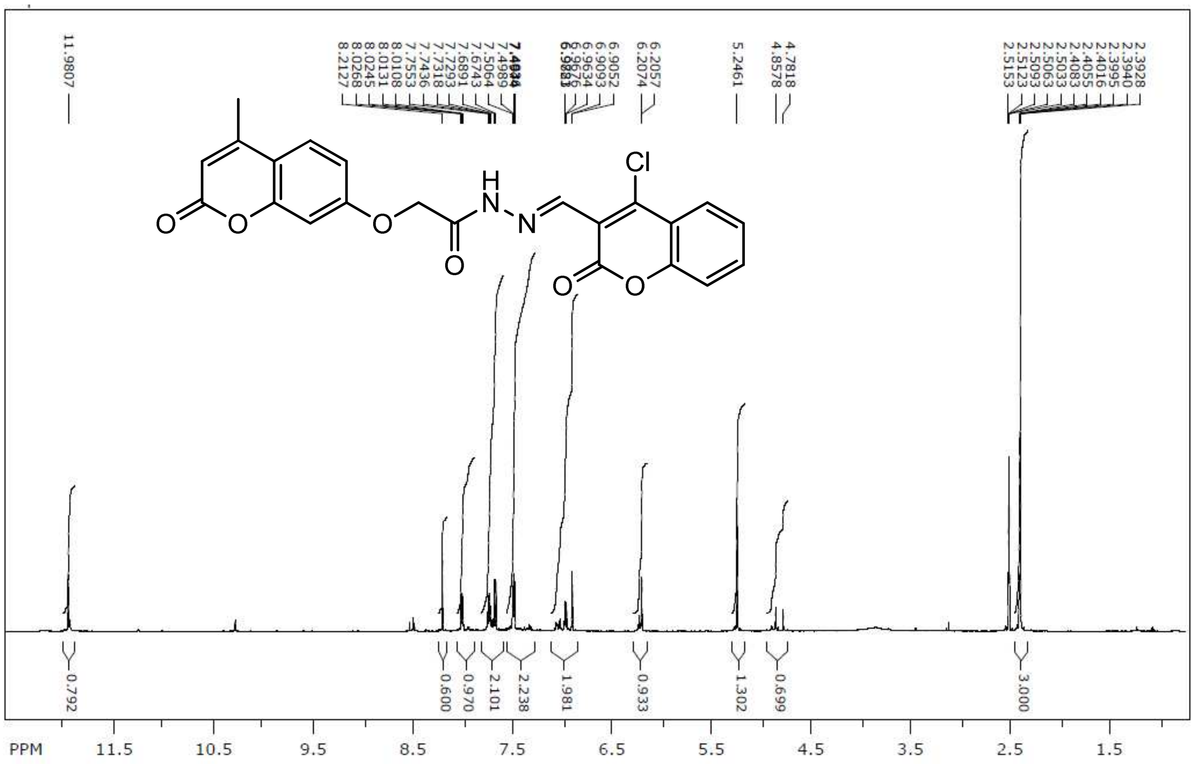

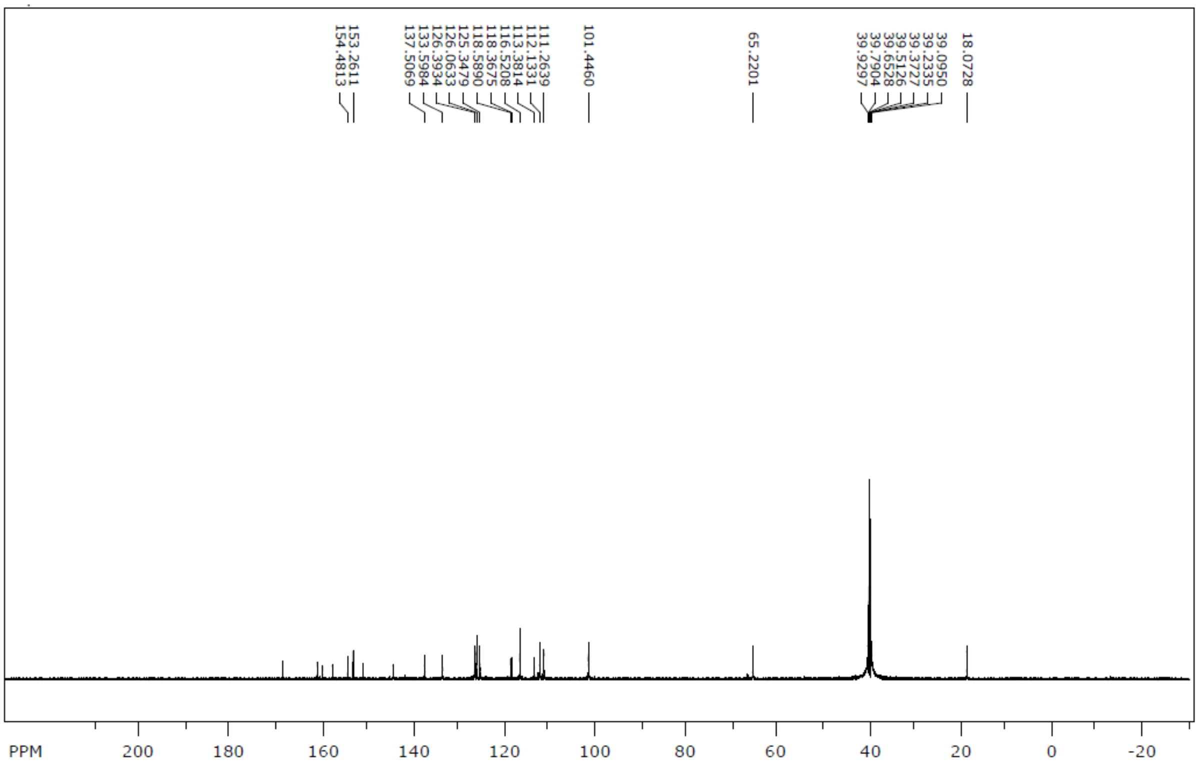

Supplement: Supplementary file 1 [file molecules-22-01482-s001.zip › Molnar S1.pdf]
